# Metabolic network analysis predicts efficacy of FDA-approved drugs targeting the causative agent of a neglected tropical disease

**DOI:** 10.1186/1752-0509-6-27

**Published:** 2012-04-27

**Authors:** Arvind K Chavali, Anna S Blazier, Jose L Tlaxca, Paul A Jensen, Richard D Pearson, Jason A Papin

**Affiliations:** 1 Department of Biomedical Engineering, University of Virginia, Charlottesville, VA, USA; 2 Department of Medicine, Division of Infectious Diseases and International Health, University of Virginia, Charlottesville, VA, USA; 3 Department of Pathology, University of Virginia, Charlottesville, VA, USA; 4 Mail: Box 800759, Health System, University of Virginia, Charlottesville, VA, 22908, USA

## Abstract

**Background:**

Systems biology holds promise as a new approach to drug target identification and drug discovery against neglected tropical diseases. Genome-scale metabolic reconstructions, assembled from annotated genomes and a vast array of bioinformatics/biochemical resources, provide a framework for the interrogation of human pathogens and serve as a platform for generation of future experimental hypotheses. In this article, with the application of selection criteria for both *Leishmania major *targets (e.g. *in silico *gene lethality) and drugs (e.g. toxicity), a method (MetDP) to rationally focus on a subset of low-toxic Food and Drug Administration (FDA)-approved drugs is introduced.

**Results:**

This metabolic network-driven approach identified 15 *L. major *genes as high-priority targets, 8 high-priority synthetic lethal targets, and 254 FDA-approved drugs. Results were compared to previous literature findings and existing high-throughput screens. Halofantrine, an antimalarial agent that was prioritized using MetDP, showed noticeable antileishmanial activity when experimentally evaluated *in vitro *against *L. major *promastigotes. Furthermore, synthetic lethality predictions also aided in the prediction of superadditive drug combinations. For proof-of-concept, double-drug combinations were evaluated *in vitro *against *L. major *and four combinations involving the drug disulfiram that showed superadditivity are presented.

**Conclusions:**

A direct metabolic network-driven method that incorporates single gene essentiality and synthetic lethality predictions is proposed that generates a set of high-priority *L. major *targets, which are in turn associated with a select number of FDA-approved drugs that are candidate antileishmanials. Additionally, selection of high-priority double-drug combinations might provide for an attractive and alternative avenue for drug discovery against leishmaniasis.

## Background

Over one billion people are infected by one or more neglected tropical diseases (NTDs) [1,2]. These diseases comprise a group of parasitic and bacterial infections that affect some of the poorest and most marginalized populations around the world, many who live on less than 1.25 USD a day [2]. Leishmaniasis is one such NTD that is endemic in 88 countries with a total of 350 million people at risk. The disease is associated with a global prevalence of 12 million cases, a yearly incidence of 1.5 to 2 million cases, and an annual mortality rate of over 59,000 deaths [3]. Caused by *Leishmania *species, the disease can manifest itself in varying clinical pathologies including visceral, cutaneous or mucocutaneous forms [3,4]. These parasites transition through different morphological stages - from flagellated promastigotes within a female phlebotomine sandfly (vector) gut to non-flagellated amastigotes in host macrophages [5]. Leishmaniasis and other NTDs tend to be overshadowed by research that is focused on malaria, tuberculosis and HIV/AIDS [6]. There may also be a lack of interest by pharmaceutical companies in pursuing drug development against NTDs due to a complicated profit motive [7]. Issues with high toxicity, life-threatening side-effects, cost, parenteral administration and emergence of resistance are drawbacks common to existing antileishmanial drugs such as sodium stibogluconate, meglumine antimoniate, pentamidine and amphotericin B [4,8]. Therefore, with limited viable treatment options and few alternatives on the pipeline, there exists an obvious need to develop radically new strategies and conceptual frameworks to identify compounds with novel antileishmanial activity.

Computational metabolic networks have been reconstructed for several pathogenic organisms [9,10], including *Leishmania major*, an agent of cutaneous leishmaniasis [11]. Implementing constraint-based modeling techniques, such as flux balance analysis (FBA), on network reconstructions has yielded valuable insight into gene essentiality and enzyme robustness under varying environmental conditions [11,12]. Accordingly, analysis of pathogen intracellular processes can be used to predict critical protein targets that when perturbed singly or in combination have adverse effects on virulence and/or growth. Here, a direct metabolic network-driven method that incorporates single gene essentiality and synthetic lethality predictions is proposed that generates a set of high-priority *L. major *targets, which are in turn associated with a select number of Food and Drug Administration (FDA)-approved drugs that are candidate antileishmanials. Subsequently, candidate drugs, which are already in clinical use for other indications, can begin to be investigated for clinical use against leishmaniasis. In addition, the selection of high-priority double-drug combinations that demonstrate superadditivity might provide for an attractive and alternative avenue for drug discovery against leishmaniasis. By integrating publicly available resources using a direct and efficient method, the approach presented here offers significant implications to future drug discovery and drug repurposing strategies against leishmaniasis and other NTDs.

## Results

### Conceptual platform of MetDP: A pipeline for prioritization of targets and FDA-approved drugs

**Figure 1:**
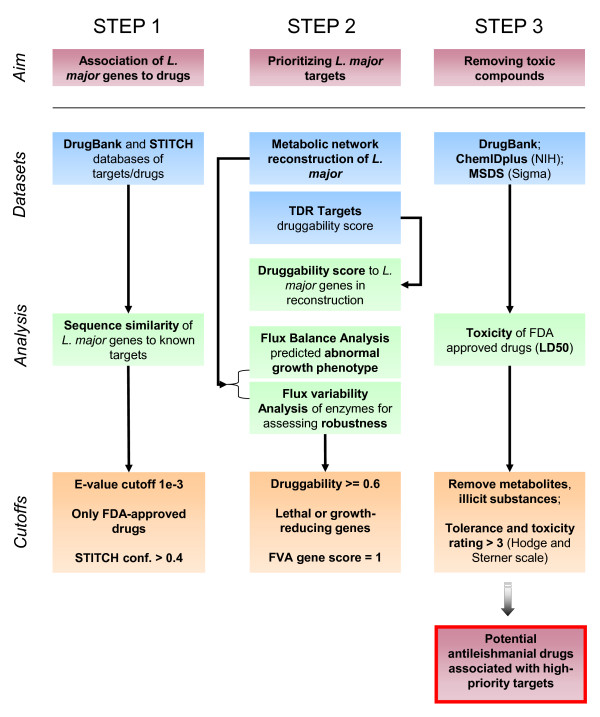
**MetDP concept**. The flowchart illustrates the conceptual framework (MetDP) for the prioritization of drug and drug targets against *L. major*. The first step deals with the association of *L. major *genes and drugs. The second step includes the prioritization of *L. major *targets and drugs using constraints such as druggability, gene essentiality and flux variability. The final step allows for the removal of toxic compounds from the prioritized list of drugs to yield a set of low-toxic candidate antileishmanials.

**Figure 2:**
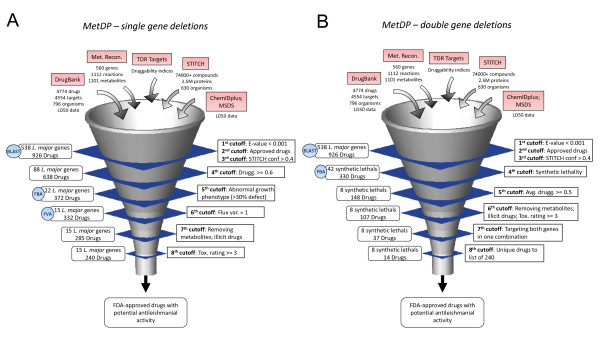
**Pipelines for prioritization of targets and drugs**. The funnel diagrams in panels **A **and **B **depict the specific datasets, tools and cutoffs used in the process of prioritization. Panel **A **presents the prioritization scheme that includes single gene deletions as a constraint, while Panel **B **presents the prioritization scheme that includes double gene deletions (or synthetic lethality) as a constraint.

The flowchart and conceptual framework for the prioritization of drug and drug targets against *L. major *(henceforth titled MetDP for Metabolic network guided Drug Pipeline) is presented in Figure 1. The specific datasets, tools and cutoffs used in the process of prioritizing effective drugs and drug targets against *L. major *are depicted in the diagram presented in Figure 2. The previously published metabolic reconstruction of *L. major *accounted for the function of 560 genes [11]. Using bioinformatics tools, a mapping was created between these 560 genes and drugs in publicly available DrugBank and STITCH databases. Using BLAST-based sequence similarity, an association was made between *L. major *genes and known targets and then transitively to drugs. Deliberately implementing an E-value cutoff and a STITCH confidence metric as well as selecting for only approved drugs resulted in 538 *L. major *genes being linked to 926 FDA-approved drugs (see Methods).

Subsequently, a set of criteria was imposed to select for those approved drugs that might demonstrate effective antileishmanial activity. Constraints were applied to both *L. major *genes (to select for high-priority targets) and drugs (to select for those with high tolerance in terms of toxicity). To arrive at suitable cutoff values, the sensitivity associated with various cutoffs used in MetDP was explored (see Additional file 1: Figures S1 and S2). In the selection of high-priority *L. major *targets, the first metric applied was a druggability score for *L. major *genes ranging from 0 (not druggable) to 1 (highly druggable). Druggability indices were obtained from the TDR Targets database. The imposition of a moderate druggability constraint of 0.6 selected for 88 *L. major *genes associated with 638 drugs (see Figure 2A). Next, computational predictions of abnormal growth phenotype (lethal or growth-reducing *in silico *gene deletions) from performing FBA on the metabolic reconstruction were also applied for the selection of priority *L. major *genes. Following sensitivity analysis (see Additional file 1: Figure S1), only those genes that when knocked out from the metabolic model yielded greater than 30% growth defect were selected. At this cutoff, 22 *L. major *genes linked to 372 drugs remained (see Figure 2A). Further, flux variability analysis (FVA) was performed on the metabolic network of *L. major *to determine flux ranges for gene-associated (or enzyme-catalyzed) reactions. The motivation to use an FVA score for genes was based on an assumption that genes linked to reactions with little variability in flux might be good targets to disrupt in the metabolic network. To elaborate, it was assumed that enzymatic reactions inflexible to take on a wide range of flux values may not necessarily be robust to network perturbations. Hence, an FVA score for genes was computed (see Methods) and used as a metric alongside the druggability index and *in silico *gene essentiality analysis to prioritize *L. major *targets. With the FVA cutoff of 1 (see Additional file 1: Figure S1 for sensitivity associated with various FVA cutoffs), the number of *L. major *genes reduced to 15 with only 332 FDA-approved drugs passing the cutoff (see Table 1 for list of targets).

**Table 1:** The list of prioritized *L. major *targets

*L. major *targets	Enzyme name	**E.C**.	Metabolic pathway(s)	Sub-cellular localization(s)†	Druggability	Growth defect*	No. of drugs
LmjF04.0580	spermidine synthase	2.5.1.16	methionine metabolism	cytosol	0.8	100% (L)	3

LmjF05.0350	trypanothione reductase	1.8.1.12	trypanothione metabolism	glycosome	0.9	100% (L)	45

LmjF05.0830	methylthioadenosine phosphorylase	2.4.2.28	methionine metabolism	cytosol	0.8	100% (L)	15

LmjF06.0650	lanosterol synthase	5.4.99.7	steroid biosynthesis	ER	0.6	100% (L)	3

LmjF11.1100	sterol 14-demethylase	1.14.13.70	steroid biosynthesis	ER	0.8	100% (L)	133

LmjF12.0280	ornithine decarboxylase	4.1.1.17	urea cycle and metabolism of amino groups	cytosol	1	100% (L)	40

LmjF13.1620	squalene monooxygenase	1.14.99.7	steroid biosynthesis	ER	0.8	100% (L)	14

LmjF18.0020	diphosphomevalonate decarboxylase	4.1.1.33	steroid biosynthesis	glycosome/cytosol	0.6	100% (L)	5

LmjF22.1360	dimethylallyltranstransferase geranyltranstransferase	2.5.1.1 2.5.1.10	steroid biosynthesis	glycosome/cytosol	0.8	100% (L)	11

LmjF25.1120	aldehyde dehydrogenase	1.2.1.3	many pathways	mitochondria	1	> 30% (GR)	28

LmjF30.3190	hydroxymethylglutaryl CoA reductase	1.1.1.34	steroid biosynthesis	glycosome/mitochondria	0.8	100% (L)	29

LmjF31.2940	squalene synthase	2.5.1.21	steroid biosynthesis	glycosome/cytosol	0.8	100% (L)	9

LmjF32.1580	phosphomannose isomerase	5.3.1.8	fructose and mannose metabolism	cytosol	0.6	100% (L)	5

LmjF33.2720	beta-ketoacyl-acyl-carrier-protein synthase I	2.3.1.41	fatty acid biosynthesis	mitochondria	0.7	100% (L)	5

LmjF35.3340	phosphogluconate dehydrogenase	1.1.1.44	pentose phosphate pathway	cytosol	0.8	100% (L)	13

* (L) Lethal, (GR) Growth-reducing; † (ER) Endoplasmic Reticulum

In parallel, high-priority synthetic lethal targets were also selected as part of the MetDP framework. The motivation for investigating synthetic lethal targets was that there may be other drugs that act on multiple *L. major *targets whose simultaneous perturbation results in growth inhibition of the parasite. Using this rationale, a synthetic lethality constraint was introduced into MetDP in order to account for those FDA-approved drugs that target genes involved in a 'non-trivial lethal' double combination (i.e. genes in the combination are individually non-essential even though the combination is essential). Using the metabolic network reconstruction of *L. major*, 56 double gene deletions (out of a total of 156,520 combinations) were previously identified as non-trivial lethal [11]. Once again, a moderate druggability constraint within the 0.3 to 0.6 range was considered: gene combinations with an average druggability index greater than or equal to 0.5. In addition, both genes in the combination needed to have moderate druggability indices of 0.5 or higher (see Figure 2B). A total of 8 non-trivial lethal double gene combinations satisfied this criterion (see Table 2 for list of synthetic lethal targets). With the synthetic lethality and druggability constraints together yielding only 8 double gene deletions, an FVA constraint was not applied in the search for high-priority synthetic lethal targets.

**Table 2:** The list of prioritized synthetic lethal targets

Gene#1	Enzyme name	Druggability	No. of drugs	Gene#2	Enzyme name	Druggability	No. of drugs	Avg. druggability
LmjF27.2050	ribonucleoside-diphosphate reductase small chain	1	13	LmjF22.1290	ribonucleoside-diphosphate reductase small chain	1	13	1

LmjF06.0860	dihydrofolate reductase-thymidylate synthase	1	76	LmjF21.1210	thymidine kinase	0.8	10	0.9

LmjF20.0100	phosphoglycerate kinase	0.8	7	LmjF24.0850	triose-phosphate isomerase	0.8	7	0.8

LmjF24.0850	triose-phosphate isomerase	0.8	7	LmjF30.3380	phosphoglycerate kinase	0.5	7	0.65

LmjF04.0960	adenylate kinase	0.5	5	LmjF34.0110	adenylate kinase	0.8	4	0.65

LmjF30.3520	S-adenosylmethionine synthetase	0.6	17	LmjF30.3500	S-adenosylmethionine synthetase	0.6	17	0.6

LmjF05.0510	F-type H+-transporting ATPase alpha chain	0.5	2	LmjF05.0500	F-type H+-transporting ATPase alpha chain	0.5	2	0.5

LmjF25.1180	F-type H+-transporting ATPase beta chain	0.5	3	LmjF25.1170	F-type H+-transporting ATPase beta chain	0.5	3	0.5

The final step in MetDP involved considerations of tolerance and toxicity in order to place emphasis on drugs that have potential clinical relevance against leishmaniasis. Drugs that are known common metabolites or chemical elements (e.g. NADH, adenine, ATP, amino acids, ethanol, iron and zinc) and present in the metabolic reconstruction were removed from further analysis. Also, any illicit drugs and controlled substances (Schedule I through V) were also removed. Second, lethal dose 50 (LD50) data were culled from DrugBank database and material safety data sheets available online, and a toxicity rating based on the Hodge and Sterner scale (see Additional file 1: Table S1) was applied. Following tolerance and toxicity analysis, the 15 single drug targets were associated with 240 drugs with a toxicity rating greater than or equal to 3 ('moderately toxic' on the Hodge and Sterner scale). Meanwhile, a total of 107 drugs were associated with the 8 non-trivial lethal gene combinations. Of these, 37 drugs were mapped to both genes involved in any one combination and 14 of them were unique when compared to the list of 240 drugs (see Figure 2B).

Consequently, upon implementation of MetDP by means of iterating through the pipeline (presented in Figures 2A and 2B), the prioritized list of *single L. major *targets included 15 metabolic genes (out of 560 genes in the metabolic reconstruction; 2.7%) while the prioritized list of *synthetic lethal *targets included 8 double-gene combinations (out of 156,520; 0.005%). Collectively, these lists of high-priority targets were associated with 254 FDA-approved drugs (out of 4329 drugs in DrugBank; 5.9%). The list of prioritized *L. major *targets is presented in Tables 1 and 2, the list of 254 FDA-approved drugs (henceforth referred to as 'Lm254') is presented in Additional file 1: Table S2, and the list of all drugs associated with the 8 synthetic lethal targets is presented in Additional file 1: Table S3.

**Figure 3:**
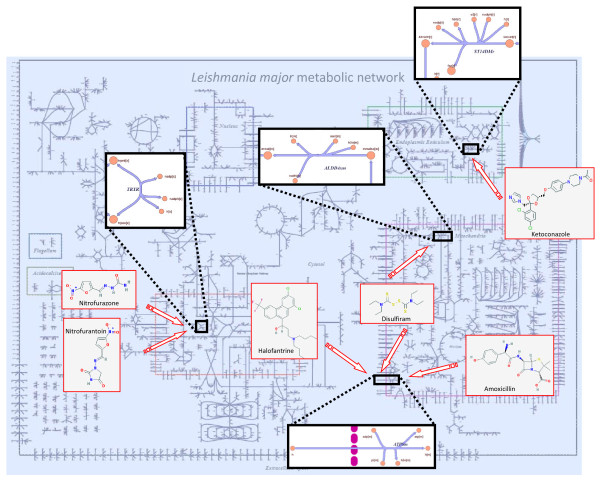
**Metabolic network analysis and the prediction of high-priority targets and drugs**. The pipeline for the prioritization of drug and drug targets against *L. major *(MetDP) is based on an underlying metabolic network. This framework enables the hypothesized description of a mechanism of action for the drugs identified. The figure highlights specific prioritized targets (singly lethal or lethal in combination) and associated drugs. Compound structures were obtained from PubChem database.

In summary, MetDP is a direct and rational method of prioritizing drugs and drug targets with a metabolic network as an underlying framework all the while providing for a possible mechanism of action for many of the drugs selected (see Figure 3).

### Target validation: Comparison of target predictions with observations from previous literature

As an initial validation, several of the genes in Table 1 were identified as proven drug targets in the literature and some have previously identified inhibitors in *Leishmania *or other related species. A particularly interesting target is *LmjF05.0350 *encoding for trypanothione reductase. In trypanosomatids, trypanothione reductase takes the place of glutathione reductase for the maintenance of intracellular redox balance [13]. Trypanothione reductase is required for the survival of *Leishmania *sp. and one important physiological role of this enzyme is defense against oxidative damage when parasites are resident inside macrophages [13]. Host glutathione reductase and parasite trypanothione reductase have mutually exclusive substrate specificities conferring an advantage for the purposes of drug targeting against the parasite enzyme [13]. Many inhibitors have been identified for trypanothione reductase, including trivalent antimony (Sb (III)) ions as recently demonstrated [14]. Also, two nitrofuran derivative compounds were experimentally shown to be effective non-competitive inhibitors of trypanothione reductase in *Trypanosoma cruzi*, a trypanosomatid organism and causative agent for Chagas disease [15]. MetDP predicted the efficacy of two nitrofurans associated with *LmjF05.0350 *namely, nitrofurazone and nitrofurantoin (see Figure 3). Other interesting targets in the prioritized list include *LmjF12.0280 *and *LmjF04.0580 *encoding for ornithine decarboxylase and spermidine synthase, respectively. Similarly to trypanothione reductase, both genes are essential to *Leishmania *sp. and model predictions are consistent with experimental observations [11]. Ornithine decarboxylase is also an important target in *Trypanosoma brucei*, another trypanosomatid organism and causative agent for human African trypanosomiasis, with a well-established inhibitor (eflornithine) [16]. See also a discussion on false negative target predictions in Additional file 1.

### Drug validation: Comparison of drug predictions to literature findings and existing high-throughput screens

As additional preliminary validation, the literature was mined to consider drugs that have been evaluated clinically against leishmaniasis (see Additional file 1: Table S4). There were nine positives in Lm254: amphotericin B [17-20], ketoconazole [17,21], fluconazole [17,22], clotrimazole [23], itraconazole [19,24], miconazole [23], terbinafine [25], metronidazole [19,26] and allopurinol [17-19]. Note that for some of these drugs, the literature is controversial with regards to their efficacy in treating leishmaniasis. From the computational network analysis, seven of the positive candidates are predicted to exclusively target *L. major *enzymes involved in steroid biosynthesis, with exceptions being ketoconazole and allopurinol. The anti-fungal ketoconazole is predicted to be associated with four *L. major *enzymes: three involved with steroid biosynthesis and one participating in fructose and mannose metabolism. And, allopurinol is associated with an enzyme involved in methionine metabolism (see Additional file 1: Table S4; see also a note on 'Dependency on confidence of interactions in DrugBank and STITCH' in Additional file 1). Previous research has identified the azole drugs ketoconazole, itraconazole, miconazole and fluconazole to be sterol 14-demethylase inhibitors that have been evaluated *in vitro *against *T. cruzi *parasites or in mouse models of *T. cruzi *infection [27]. Additionally, in the fungal species *Candida albicans*, clotrimazole and terbinafine have been shown to be inhibitors of sterol 14-demethylase and squalene monooxygenase, respectively [28,29]. These references from other organisms serve as a check for some of the computationally predicted links in MetDP between *L. major *genes and drugs.

There were also four false negative results: imiquimod [20], paromomycin [17,19,20], pentamidine [17-20] and sodium stibogluconate [17-21]. Imiquimod is a modulator of the innate immune response [30], and paromomycin is an aminoglycoside antibiotic that binds to the aminoacyl decoding site of the ribosomal 16S RNA [31,32]. Since both imiquimod and paromomycin do not primarily target *L. major *proteins, their exclusion from Lm254 was obvious. However, the reasons for exclusion of all four drugs from Lm254 were examined in further detail (see Additional file 1). Hence, nine out of thirteen (69.2%) clinically relevant drugs for leishmaniasis were present in Lm254. Three other drugs used clinically against leishmaniasis (meglumine antimoniate [17-20], miltefosine [20,33] and sitamaquine [19,20,34]) were not considered in the analysis as they were not present in DrugBank.

Additionally, several high-throughput drugs screens against *in vitro *growth of *Leishmania *and *Trypanosoma *species have been recently published [35-38]. In particular, one study by Sharlow et al. screened 196,146 compounds at 10 μM against *L. major *promastigotes [37]. A total of 187 FDA-approved drugs that were also in DrugBank database overlapped with the set of 196,146 compounds; 68 out of 187 were present in Lm254. Of these 68 drugs, seven (pimozide, furazolidone, perphenazine, bifonazole, disulfiram, clotrimazole and floxuridine) were active as primary hits in the high-throughput screen, and the remaining 61 were classified as inactive as they did not meet the 50% inhibition threshold when evaluated at 10 μM. Furthermore, during assay optimization and validation screening in the same study, two additional drugs not included in the list of 68, tamoxifen and mycophenolic acid, were also deemed to be primary hits [37]. These two drugs were also included in Lm254. A second high-throughput screening (HTS) study for inhibitors cytotoxic to bloodstream form *T. brucei *tested 2,160 FDA-approved drugs, bioactive compounds and natural products and produced 35 hits when assayed at a concentration of 1 μM [38]. One drug, paclitaxel, which was included in the list of 35 hits was also present in Lm254. Paclitaxel was not included in the set of 68 drugs evaluated in the Sharlow et al. study. In previous literature, paclitaxel along with IFNγ was shown to induce killing of *L. major *infected murine macrophages [39]. In summary, from comparison to HTS data, 10 out of 71 (14.1%) drugs from Lm254 that were experimentally evaluated via HTS were classified to be potential antileishmanial hits.

Taken together, the inclusion of all clinically relevant candidates and existing HTS data yields a total of 18 drugs (7.1% of Lm254) as having potential for antileishmanial activity (see Additional file 1: Table S2 for the list of 18 drugs). The HTS data and data on drugs that have been used clinically against leishmaniasis account for only 83 out of 254 drugs. Any drugs in Lm254 not previously evaluated against *L. major *parasites (*in vitro *and/or *in vivo*) serve as prime candidates for future investigation into the discovery of antileishmanial compounds.

### *In vitro *experimental evaluation of halofantrine

**Figure 4:**
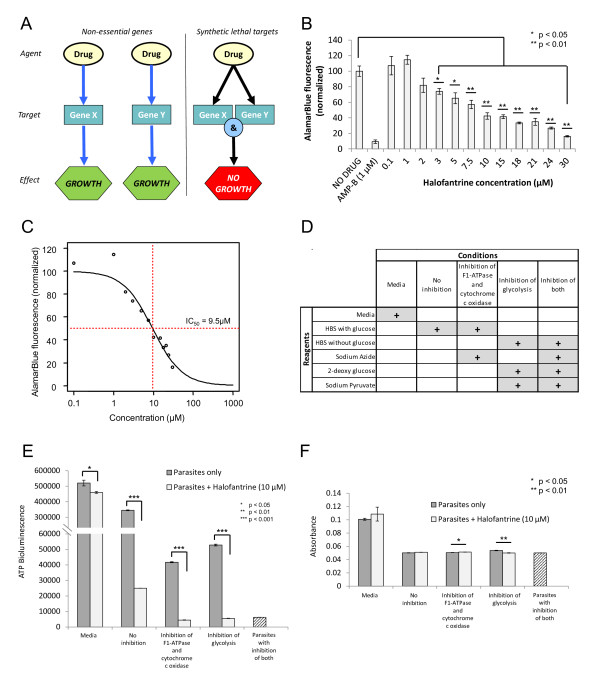
**Synthetic lethality and experimental evaluation of halofantrine against *L. major *promastigotes**. Synthetic lethality analysis highlights an interaction between genes in the combination; a simultaneous knockout of genes in the combination causes a perturbation in the functioning of associated reactions in the network, thereby leading to an adverse effect on biomass production. A similar effect on biomass would not occur if the genes in the combination were knocked out individually. Panel **A **illustrates the concept of a drug acting on multiple targets (that are synthetically lethal) in order to inhibit growth of *L. major*. The effect of halofantrine evaluated at different concentrations against *L. major *promastigotes is presented in Panel **B**. The y-axis indicates alamarBlue fluorescence normalized to the 'No Drug' control. AMP-B refers to Amphotericin B, which is used as a positive control in the assay. In Panel **C**, a four-parameter log-logistic regression was performed on the concentration response data to compute the IC_50 _for halofantrine against *L. major *promastigotes. Panel **D **provides a setup for the experimental conditions and corresponding reagents used for the ATP bioluminescence assay to determine the effects of halofantrine on ATP levels in *L. major*. The results of the ATP bioluminescence assay are presented in Panel **E**. Parasites were incubated with or without halofantrine at 10 μM and in the presence of mitochondrial and/or glycolytic ATP blocks for 2 hours. The absorbance was monitored at 18 hours and the results are shown in Panel **F**. All error bars indicate standard error. Statistical significance in panels **B **and **E **was determined using a one-tailed Student's t-test, while statistical significance in panel **F **was determined using a two-tailed Student's t-test. As an additional note, the absorbance measurements in panel **F **between parasites incubated with and without halofantrine are significant for two conditions. However, this significance is attributable to the precision of the microplate reader rather than any meaningful biological implications regarding variations in absorbance. In Panels **E **and **F**, the condition where parasites are incubated with both mitochondrial and glycolytic ATP blocks is displayed with a dashed bar as it serves as a reference. In this particular case, halofantrine was not added.

The next step involved the *in vitro *evaluation of candidate antileishmanials as prioritized using MetDP. Halofantrine, an antimalarial agent, was one of 14 drugs that was selected via synthetic lethality analysis (see Figure 4A), and was experimentally evaluated for antileishmanial activity against *L. major *promastigotes. This drug was not included in the HTS data that were used to validate the selection of high-priority drugs. Halofantrine showed noticeable antileishmanial activity in a concentration response assay (see Figure 4B). When compared to the 'No Drug' control, the effect of halofantrine at 3 μM and higher concentrations was statistically significant (*p *< 0.05). The IC_50 _for halofantrine against *L. major *promastigotes was calculated to be approximately 9.5 μM (Figure 4C).

Originally, from the DrugBank database, halofantrine was associated with vacuolar ATP synthase catalytic subunit A from *Plasmodium falciparum*. Upon implementation of the MetDP pipeline, halofantrine was associated with the following synthetic lethal pairs: *LmjF05.0500 *&*LmjF05.0510*, genes encoding for F-type H+-transporting ATPase alpha chain, and *LmjF25.1170 *&*LmjF25.1180*, genes encoding F-type H+-transporting ATPase beta chain. While the exact mechanism of action for halofantrine in *L. major *requires further investigation, the model predictions for halofantrine represent testable hypotheses. Towards that end, an ATP bioluminescence assay was performed to further investigate the effect of halofantrine on ATP levels in *L. major *promastigotes.

As Figure 4D demonstrates, parasites were incubated with or without 10 μM halofantrine in various conditions as follows: (a) media, (b) HEPES-buffered saline (HBS) for 'No inhibition', (c) HBS with sodium azide for 'Inhibition of F1-ATPase and cytochrome *c *oxidase', (d) HBS without glucose, but supplemented with 2-deoxy-D-glucose and sodium pyruvate for 'Inhibition of glycolysis', and (e) HBS without glucose, but supplemented with sodium azide, 2-deoxy-D-glucose and sodium pyruvate for 'Inhibition of both'. In the assay, sodium azide was used to inhibit mitochondrial oxidative ATP generation [40]. Further, 2-deoxy-D-glucose (a glucose analog and competing substrate for hexokinase) was used along with sodium pyruvate in glucose-free buffer to inhibit glycolytic ATP synthesis [40]. In Figure 4E, when the parasites were subject to 'No inhibition,' halofantrine caused a significant reduction in ATP levels. Figure 4E also shows that ATP levels in parasites under 'Inhibition of F1-ATPase and cytochrome *c *oxidase' dropped significantly when halofantrine was added. Likewise, ATP levels in parasites under 'Inhibition of glycolysis' dropped significantly when halofantrine was added. In both these instances, ATP levels dropped to a level comparable to parasites subject to the simultaneous inhibition of glycolysis and F1-ATPase/cytochrome *c *oxidase ('Inhibition of both'). Hence, the ATP bioluminescence assay suggests that halofantrine has an effect on either one or both of glycolytic and mitochondrial oxidative ATP generation mechanisms. Future experimental efforts can be directed at more precise characterization of the underlying mechanism of action of the drug and elucidating other potential metabolic targets of halofantrine in *L. major*. Correspondingly, absorbance was monitored to make certain that the effects that were seen with regard to ATP levels were not a function of variations in cell count across the various conditions (Figure 4F). Finally, although halofantrine is known to be associated with cardiotoxicity [41], its potential as an antileishmanial agent should be investigated in more detail.

### Model-guided drug combinations as an alternative strategy against leishmaniasis

With the ability to computationally simulate synthetically lethal gene deletions, there exists an opportunity to predict and prioritize multiple combination drug therapies that may be superadditive/synergistic. A potential advantage of superadditive drug combinations is overcoming toxicity or side-effects linked to high doses of individual drugs needed to establish the same inhibitory effect as the combination [42]. One strategy of prioritizing for clinically-relevant drug combinations was to selectively focus on one drug that demonstrated excellent antileishmanial activity *in vitro *and was associated with low-toxicity. All combinations involving that particular drug could be prioritized for experimental analysis. Selection of such high-priority drug combinations through the power of network analysis can augment single compound discovery strategies.

Since disulfiram was previously shown to have a sub-micromolar IC_50 _[37] (see also dose response data for disulfiram in Additional file 1: Figure S7), the goal was to identify superadditive drug combinations that involve disulfiram. Marketed as Antabuse, disulfiram has an LD50 of 8600 mg/kg (rat; oral) and is used in the treatment of alcoholism. At a moderate STITCH confidence of greater than 0.4, none of the 8 high-priority synthetic lethal gene predictions were associated with disulfiram. Therefore, the search criterion was expanded to include gene-drug interactions at a lower STITCH confidence (greater than 0.15; see Methods for mapping between *L. major *genes and drugs; see also Additional file 2 for a list of initial gene-drug associations).

**Figure 5:**
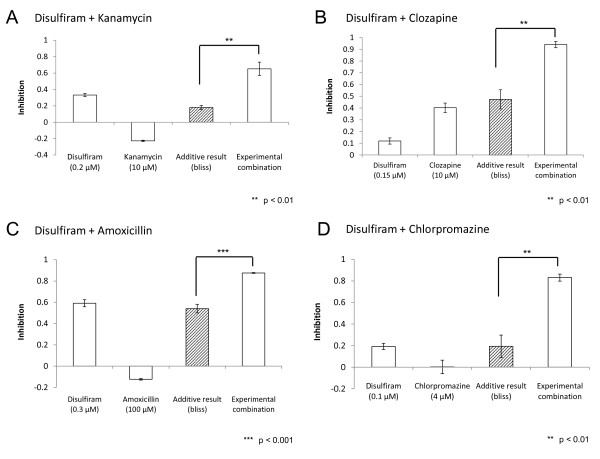
**Drug combinations involving disulfiram**. Disulfiram along with kanamycin, clozapine, amoxicillin and chlorpromazine are all predicted to be individually effective antileishmanial agents because they all act on both genes in a synthetic lethal pair. However, they also have the potential to act synergistically to produce growth inhibition in *L. major*. Therefore, for predictions of model-guided superadditivity, it is assumed that the interactive relationship present between genes in a synthetic lethal deletion would translate directly to any associated drug combinations. In panels **A**, **B**, **C **and **D**, the results of one concentration profile of disulfiram + kanamycin, disulfiram + clozapine, disulfiram + amoxicillin and disulfiram + chlorpromazine as evaluated against *L. major *promastigotes are presented, respectively. The theoretical additivity bar was computed using the bliss additivity metric upon comparing the effects of the individual drugs. A two-sample t-test comparing two means was used to determine statistical significance between the theoretical and experimental combinations. Concentrations are provided in parentheses. Error bars signify standard error. The y-axis indicates fractional experimental effect of inhibition or growth relative to "No Drug" control (0 equals no inhibition, 1 equals max inhibition). All data were generated at 48 hours post addition of alamarBlue dye.

With the relaxation of the STITCH confidence constraint, one pair of high-priority synthetic lethals was associated with disulfiram: *LmjF25.1170 *&*LmjF25.1180*, genes encoding F-type H+-transporting ATPase beta chain. After tolerance and toxicity analysis, both genes were associated with the same 16 drugs (one of them being disulfiram; see Additional file 1: Table S5). Since the 16 drugs are predicted to act on both genes in a synthetic lethal pair, they are consequently predicted to be effective individually as well. All possible drug combinations involving disulfiram (a total of 15) were then selected. Combinations of disulfiram with six other drugs (kanamycin, clozapine, amoxicillin, chlorpromazine, doxycycline and isoniazid) were experimentally evaluated against *L. major *promastigotes using the alamarBlue assay. Figure 5 demonstrates the superadditive nature of the experimental combinations of disulfiram with kanamycin, clozapine, amoxicillin, and chlorpromazine. In all four cases, the experimental combinations had a significantly greater inhibitory effect on parasite growth as compared to the calculated additive results (see Methods for calculations on additivity; only one representative concentration profile is displayed in Figure 5; see also Additional file 1: Figures S8, S9, S10 and S11 for data on other concentrations tested). Combinations of disulfiram with doxycycline and isoniazid did not show statistically significant superadditivity (data not shown).

Two of the combinations that demonstrated superadditivity involved antibiotics - kanamycin is an aminoglycoside antibiotic and amoxicillin is a common β-lactam antibiotic. The other two combinations involved antipsychotics - clozapine and chlorpromazine are used in the treatment of symptoms associated with schizophrenia. Of the four combinations, disulfiram + amoxicillin had the best overall toxicity rating. Moreover, only clozapine and chlorpromazine demonstrated inhibitory effects on *L. major *promastigotes when tested individually (see Additional file 1: Figures S9 and S11). Kanamycin and amoxicillin did not demonstrate any inhibitory effect at the concentrations tested (see Additional file 1: Figures S8 and S10).

Interestingly, along with disulfiram, kanamycin and chlorpromazine are also present in Lm254. In the future, evaluating two-drug combinations where both drugs are predicted to be effective individually by acting on single lethal or growth-reducing targets may have certain advantages. Based solely on synthetic lethality analysis, in the event that the parasite develops resistance to one of the drugs (in a combination) due to mutations in an enzyme/gene product encoded by a single gene in a synthetic lethal deletion, the overall drug combination would be predicted to be ineffective. However, by considering only those drug combinations where the individual drugs involved are also independently effective antileishmanial agents, this limitation can be potentially overcome. If the organism becomes resistant to one of the drugs during infection, there is a potential compensatory mechanism in place for the other drug to inhibit growth. This redundancy may serve to prevent or delay the onset of resistance [43].

## Discussion

Recent literature has described the applicability of metabolic network analysis towards target identification and drug discovery in general. One study (targetTB) used a variety of network analyses and bioinformatics-based sequence/structural assessments to predict a list of targets against *Mycobacterium tuberculosis *[44]. By prioritizing targets using a layered approach, proteins that did not pass sequential cut-offs were filtered out [44]. Another study implemented network analysis and molecular docking simulations to identify small molecule inhibitors of type II fatty-acid biosynthesis enzymes in *Escherichia coli *and *Staphylococcus aureus *and experimentally evaluated the computational predictions [45]. FBA was also used to simulate drug synergy effects by assigning synergy scores to combinations of enzymes that would inhibit growth of *E. coli *in various environmental conditions [42]. Network reconstructions of *P. falciparum *and *Vibrio vulnificus *have also guided the prediction of drug targets, and investigational compounds were experimentally evaluated against these pathogens [9,46].

Here, a novel pipeline for the prioritization of drugs and drug targets is presented that is in many ways distinct from approaches previously developed. Instead of restricting the search to compounds that may only target non-human proteins or have computed Tanimoto similarity to important metabolites, MetDP undertook a more expansive strategy in making use of protein-compound interactions present in both DrugBank and STITCH resources. Importantly, a clear majority of the approved drugs target human proteins as opposed to proteins in any other single organism (e.g. in DrugBank). Therefore, rather than avoiding the selection of pathogen targets that are similar to human proteins, MetDP explicitly sought out such targets in order to create a link between *L. major *genes and drugs that are already approved. The side-effects caused by any of the drugs in the prioritized list that act on related human targets are relatively well known.

In addition, by restricting the search to only FDA-approved drugs and eliminating investigational drugs from the selection process, major regulatory hurdles that novel compounds are subject to before approval for widespread clinical use are bypassed. For instance, on average, it can take more than 15 years and over 800 million USD to bring a single novel drug to market [47]. Moreover, only 20 to 30 new drugs are approved by the FDA annually [47]. Instead, drugs already approved for clinical use have known pharmacokinetics and toxicity/safety profiles (many approved drugs have met superior safety standards with phase IV post-market safety surveillance) [47]. Therefore, by demonstrating novel antileishmanial activity for a drug already in clinical use for another indication, a candidate drug or perhaps combinations of drugs can begin to be effectively repurposed by being evaluated in phase II clinical trials and bypassing approximately 40% of the overall cost that it takes to bring a novel drug to market [47]. Besides, an FDA-approved drug with demonstrated antileishmanial activity can also be prescribed off-label, thus making it accessible to patients urgently in need of an alternative treatment option.

## Conclusions

Ultimately, metabolic reconstructions provide a framework for the interrogation of human pathogens and serve as a platform for generation of future experimental hypotheses. This metabolic network-driven approach identified 15 *L. major *genes as high-priority targets, 8 high-priority synthetic lethal targets, and 254 FDA-approved drugs as potential antileishmanial agents. As experimental validation, the antimalarial drug halofantrine was shown to have noticeable antileishmanial activity. Moreover, through the ATP bioluminescence assay, halofantrine affected either one or both of glycolytic and mitochondrial ATP generation mechanisms. Additionally, synthetic lethal predictions from the metabolic network aided in the selection of drug combinations with potential for superadditivity. For proof-of-concept, double-drug combinations were evaluated *in vitro *against *L. major *and four novel superadditive combinations involving the drug disulfiram were discovered. Selection of such high-priority double-drug combinations guided by metabolic network analysis might provide for an attractive and alternative avenue for drug discovery against infectious diseases like leishmaniasis.

A rational method of prioritizing drugs and drug targets with a metabolic network as an underlying framework also provides for a possible mechanism of action for many of the drugs selected (e.g., the network model enables the analysis of what biosynthetic pathways are inhibited when a particular enzyme's function is pharmacologically inhibited). Surely the mechanism of action is only predicted and needs to be validated with follow-on experimentation. However, given a drug and potential target, a starting experimental hypothesis is provided. A network-driven approach guiding the selection of prioritized drugs can be immensely advantageous in terms of cost and efficiency in the beginning phases of drug discovery and can offer significant implications to future drug repurposing strategies against a variety of NTDs.

## Methods

### Datasets used

#### *L. major* reconstruction

The previously published metabolic reconstruction of *L. major *was the first of its kind for a protozoan. It accounted for 560 genes, 1112 reactions, and 1101 metabolites spanning eight unique sub-cellular localizations [11].

#### DrugBank

The DrugBank knowledgebase (version 2.5; http://www.drugbank.ca/) included 4774 drugs that were classified into several categories namely, (a) small molecule, (b) biotech, (c) approved, (d) experimental, (e) nutraceutical, (f) illicit, and (g) withdrawn. These drugs were linked to 4554 peptide/protein and non-peptide/non-protein drug targets.

#### STITCH

The STITCH database (version 2.0; http://stitch.embl.de/) comprised over 74000 small molecules and 2.5 million+ proteins in 630 organisms. The STITCH database includes many potentially important off-targets to particular compounds (derived from experimental data, other curated databases or text mining) in addition to the primary known targets, and all protein-compound interactions in STITCH have an associated confidence metric. It should be noted that *L. major *was not present in the list of organisms whose targets are explicitly included in STITCH.

#### TDR Targets

Druggability indices for *L. major *genes were downloaded from the TDR Targets database (version 3; http://tdrtargets.org/). The druggability index was calculated using a combination of approaches including sequence similarity to known biological targets associated with FDA-approved drugs from curated databases (e.g. DrugStore and StARLITe), and a sequence-based Bayesian learning algorithm, among other methods [48]. The index ranges between 0 (not druggable) and 1 (highly druggable). In all, 1598 *L. major *genes have a corresponding druggability index in the TDR Targets database. Of these, 261 are accounted for in the metabolic reconstruction.

#### DEA controlled substances

The list of controlled substances by Schedule was downloaded from the Drug Enforcement Administration (DEA) Office of Diversion Control database (http://www.deadiversion.usdoj.gov/schedules).

### Tools and programs used

#### BLAST

A BLAST+ v.2.2.24 executable was downloaded from the NCBI FTP site (ftp://ftp.ncbi.nih.gov/blast/executables/blast+/). The program *makeblastdb *was implemented on both DrugBank and STITCH target protein sequences. The amino acid sequences for all 560 *L. major *genes from the metabolic reconstruction were obtained from GeneDB (version 2.1; http://www.genedb.org/genedb/leish). Subsequently, the program *blastp *was used to query 560 *L. major *sequences against both DrugBank and STITCH target databases.

#### FBA/FVA/COBRA toolbox

Flux Balance Analysis (FBA) and Flux Variability Analysis (FVA) were implemented using the COBRA toolbox (version 1.3.3; http://opencobra.sourceforge.net/openCOBRA/Welcome.html) in MATLAB. FBA and FVA have been extensively implemented on metabolic networks of various organisms ranging from prokaryotes to eukaryotes [12].

### Mapping between *L. major *genes and drugs in DrugBank and STITCH

First, a BLASTP search was performed with 560 *L. major *genes from the metabolic reconstruction (query sequences) against 4538 target proteins in DrugBank (target sequences). An E-value cutoff was set at 0.001, and repetitious links between *L. major *genes and DrugBank target proteins were removed. At this first cutoff, 440 *L. major *genes were linked to 1116 DrugBank target proteins and transitively to 1129 drugs. The list of drugs was refined to focus on only FDA-approved drugs by removing experimental and withdrawn drugs, which resulted in 313 *L. major *genes being linked to 257 FDA-approved drugs.

In parallel, a similar approach with some variations was undertaken with the STITCH database. In order to be as expansive as possible in making initial associations between *L. major *genes and drugs, both DrugBank and STITCH resources were used. A BLASTP search was performed between the 560 *L. major *genes and 2.5 million+ proteins (2,590,259) to associate *L. major *genes with the proteins in STITCH. Protein sequences were derived from a related STRING database (http://string-db.org/). Again, an E-value cutoff of 0.001 was chosen and repetitious associations between *L. major *genes and STITCH proteins were removed. At this cutoff, all 560 genes were associated with 131,250 protein targets from STITCH.

Subsequently, the STITCH compound-protein interaction dataset was refined to only consider compounds that were FDA-approved drugs (as classified in the DrugBank database). PubChem compound identifiers were used to map drugs in DrugBank with compounds in STITCH. Only 1337 of the 1466 approved drugs in DrugBank had readily available PubChem compound identifiers (and a few had similar PubChem IDs). In all, 1330 drugs with unique DrugBank IDs mapped to 1305 unique compounds in the STITCH dataset. Next, the STITCH database was further reduced to consider protein targets strictly present in the list of 131,250 unique protein targets that made the BLAST cut. Finally, the STITCH dataset was also constrained to only include medium confidence and higher interactions (STITCH scores greater than 0.4). The newly constrained STITCH dataset along with the BLAST results were used to link 538 *L. major *genes to 905 FDA-approved drugs. At this stage, results from DrugBank and STITCH were merged for further refinement and analysis. The merged list linked 538 *L. major *genes to 926 FDA-approved drugs (see Additional file 2).

### Generating an FVA score for genes

Flux variability analysis (FVA) allows for ascertaining the complete range of numerical values for every flux in the biochemical network given an optimal value of the objective [49]. FBA computes only one possible optimal reaction network state. Therefore, the flux distribution resulting from FBA (i.e. the enumeration of all possible fluxes in the network) is only one of many feasible solutions that can exist for the same optimal value of the cellular objective. Enzymatic reactions associated with small ranges of flux values may not necessarily be robust to various kinds of network perturbations. Therefore, an FVA score for genes was developed based on an assumption that genes linked to reactions with little flux variability might be good targets to disrupt in the metabolic network.

Step 1: Conferring weights on reactions based on FVA

From the FVA output, the computed flux ranges [MaxFlux - MinFlux] for every reaction in the network were used to generate an FVA reaction score (r) between 0 (low) and 1 (high).

$$r_{i}=1-\frac{MaxFlux_{i}-MinFlux_{i}}{2*\text{max}\left(\left|MaxFlux_{i}\right|,\left|MinFlux_{i}\right|\right)}$$

This scoring method gives a high score to reactions with little variability in flux. For example, reactions that spanned the entire flux range [-999999, 999999] would receive a score of 0, while reactions with no change in flux would receive a score of 1.

Step 2: Evaluating the contributions of genes in gene-protein-reaction (GPR) relationships

Separately, GPR relationships, formulated as Boolean logic statements in the published reconstruction of *L. major *[11], were used to confer weights on genes accounted for in the network. The weights were calculated as follows:

$$c_{g_{i},r_{k}}=\frac{PT_{i}^{k}+AF_{i}^{k}-PF_{i}^{k}-AT_{i}^{k}}{N_{states}}$$

*N_states _*refers to the number of possible states for a given GPR, which is calculated as 2^n ^with *n *being the number of genes in a GPR. The GPR was evaluated to be TRUE or FALSE for every state. For a given reaction *k *with a GPR, the presence (P) or absence (A) of a particular gene *i *and the TRUE (T) or FALSE (F) outcome of the GPR were tabulated as matches (*PT_i _*and *AF_i_*) and mis-matches (*PF_i _*and *AT_i_*). Explicitly, for a given reaction *k*, 'PT_i_' refers to the number of gene states (out of 2^n^) where a particular gene *i *is present, and the GPR is evaluated to be TRUE. 'AT_i_' refers to the number of gene states where gene *i *is absent, and the GPR is evaluated to be TRUE (e.g. due to the presence of isozymes). 'PF_i_' refers to the number of gene states where a particular gene *i *is present, and the GPR is evaluated to be FALSE (e.g. subunits of a larger protein complex that need to be present together). Finally, 'AF_i_' refers to the number of gene states where a particular gene *i *is absent, and the GPR is evaluated to be FALSE. The weights (c_g,r_) were calculated by totaling up matches (counted positively) and mis-matches (counted negatively) and dividing by the number of possible states for a given GPR. Hence, a gene-reaction matrix (C) was constructed with elements of the matrix representing weights for genes linked to corresponding reactions.

Step 3: Conferring weights on genes based on FVA

Next, the FVA reaction scores (r_i_) were multiplied in with the gene weights from the GPR (c_g,r_) to yield an FVA-gene matrix with the same dimensions as C. The maximum value for genes across all reactions was considered as the FVA score for a gene (value resided between 0 and 1).

### Calculations of additive results

The additive results in Figure 5 (i.e. the non-synergistic expectation of the experimental results of the individual drugs) were computed based on the bliss additivity metric [50]:

E(x,y)=E(x)+E(y)-E(x)E(y)

In the equation above, 'E' refers to the fractional effect between 0 and 1, while 'x' and 'y' are the concentrations of the two drugs in the experimental combination. Additionally, error was propagated using the formula [50]:

$$\delta E_{1,2}=\delta E_{1}+\delta E_{2}+\left(\delta E_{1}/E_{1}+\delta E_{2}/E_{2}\right)*E_{1}\left(x\right)*E_{2}\left(y\right)$$

Here, E_1_±δE_1 _refers to the effect of drug 1 and E_2_±δE_2 _refers to the effect of drug 2.

Subsequently, a two-sample t-test was performed to compare the additive result and the experimental combination. A t-score test statistic was generated using the formula:

$$t=\frac{\left[{\bar{x}}_{1}-{\bar{x}}_{2}\right]}{\sqrt{\frac{{s_{1}}^{2}}{n_{1}}+\frac{{s_{2}}^{2}}{n_{2}}}}$$

Here, ${\bar{x}}_{1}$ and ${\bar{x}}_{2}$ refer to the means of the samples, s_1 _and s_2 _refer to the standard deviations of the samples, and n_1 _and n_2 _refer to the number of samples. One-tail p-value was subsequently determined by referring to a t-table (degrees of freedom computed as follows: n_1 _+ n_2 _- 2).

### Experimental methods

#### Materials

Black flat-bottom 96-well microtiter plates were purchased from Fisher Scientific (http://www.fishersci.com) and used in all alamarBlue experiments. White flat-bottom 96-well microtiter plates also purchased from Fisher Scientific were used in all bioluminescence experiments. alamarBlue was purchased from Invitrogen (http://www.invitrogen.com). CellTiter-Glo was purchased from Promega (http://www.promega.com). All compounds used in this study were purchased from Sigma-Aldrich (http://www.sigmaaldrich.com/). Compounds were solubilized in dimethyl sulfoxide (DMSO) or water.

#### Parasite cultures

Previously published protocols on culturing *L. major *[37] were adhered to in this study. *L. major *promastigotes and protocol for preparing media were kindly provided by Mary E. Wilson and Melissa A. Miller, University of Iowa. Parasites in complete HOMEM (see Additional file 1) were cultured in 25 cm^2 ^plastic tissue culture flasks with sealed or vented caps and maintained at 26°C.

#### alamarBlue assay

The assay was conducted in accordance with previously established protocols [51-53]. Briefly, promastigotes were diluted to 1 × 10^6 ^cells/mL, and in a black flat-bottom 96-well microtiter plate, 180 μL of suspension was incubated with varying concentrations of drugs (singly or in combination) in triplicate. Specifically, 160 μL of parasite samples were first seeded in triplicate. Next, sample wells were topped off with 20 μL of media + drug(s) (ratio altered to achieve specific concentrations of drug(s)) such that the total volume equaled 180 μL. Heat-killed parasite samples (incubated at 60°C for 20 minutes) prepared at 1 × 10^6 ^cells/mL were also seeded in triplicate (160 μL of sample + 20 μL of media) to serve as a positive control. Amphotericin B at 1 μM also served as another positive control. If DMSO was used to solubilize the drug(s), three wells with the highest relevant concentration of DMSO were included in the plate as a negative control. Additionally, three wells were seeded with 180 μL of media alone. The plate was incubated at 26°C for 24 hours at which time point 20 μL of alamarBlue dye was added to all control and experimental wells. Using a Gemini EM Microplate Spectrofluorometer, fluorescence was monitored at excitation/emission wavelengths of 544 nm/590 nm at 24 and 48 hours post addition of dye to wells. Calibration data for alamarBlue assay is provided in Additional file 1: Figures S4, S5 and S6.

#### Bioluminescence assay

The protocol for the bioluminescence assay was modified from [40]. Parasites at 8 × 10^6 ^cells/mL were incubated in culture medium or various buffers for 2 hours at 26°C either alone or in the presence of 10 μM halofantrine. Mitochondrial oxidative ATP generation was inhibited by incubating the parasites in HBS buffer with glucose plus 20 mM sodium azide, an inhibitor of F1-ATPase and cytochrome *c *oxidase from complex IV [40]. Glycolytic ATP generation was inhibited by incubating the parasites in glucose-free HBS buffer plus 5 mM 2-deoxy-D-glucose, a competitor with glucose for hexokinase binding, and 5 mM sodium pyruvate [40]. In a white opaque flat-bottom 96-well microtiter plate, 25 μL of parasite samples from each condition were seeded in triplicate. Heat-killed parasite samples (incubated at 60°C for at least 20 minutes) prepared at 8 × 10^6 ^cells/mL were also seeded in triplicate. Additionally, three wells were seeded with 25 μL of media alone. Subsequently, 25 μL of CellTiter-Glo was added to all control and experimental wells. The plate was incubated in the dark at 26°C for 10 minutes. Luminescence was monitored using a FLUOstar Optima plate reader (BMG Labtech). For absorbance measurements, 100 μL of control and experimental samples were seeded in triplicate at the 18 hour time point. The plate was immediately transferred to a Tecan infinite200 Pro microplate reader, and absorbance was monitored at 600 nm. Calibration data for the bioluminescence assay is provided in Additional file 1: Figures S12 and S13.

## Competing interests

The authors declare that they have no competing interests.

## Authors' contributions

AKC performed the computational and experimental analysis. AKC, ASB and JLT performed the experiments. PAJ helped with the computational analysis. RDP assisted with the interpretation of the experimental data. AKC and JP conceived and designed the study. All authors read and approved the final manuscript.

## Supplementary Material

Additional file 1 **In this supplement, additional experimental data, analysis and network characteristics are presented that are not already described in the main article **[11,17-22,54]. Click here for file

Additional file 2 **In this supplement, initial gene-drug associations, various metric scores for *L. major *genes, synthetic lethal predictions, toxicity ratings for drugs, and list of drugs removed from MetDP analysis are presented**. Click here for file
