# Supplementary material for: Metabolic network analysis predicts efficacy of FDA-approved drugs targeting the causative agent of a neglected tropical disease
Source: BMC Syst Biol. 2012 Apr 27;6:27. doi: 10.1186/1752-0509-6-27 (PMC3388006; doi:10.1186/1752-0509-6-27)
Supplement: Additional file 1 — In this supplement, additional experimental data, analysis and network characteristics are presented that are not already described in the main article [11,17-22,54]. [file 1752-0509-6-27-S1.PDF]

Metabolic network analysis predicts efficacy of FDA-approved drugs targeting the causative agent of a neglected tropical disease

Arvind K. Chavali[1], Anna S. Blazier[1], Jose L. Tlaxca[1], Paul A. Jensen[1], Richard D. Pearson[2,3] and Jason A. Papin[1,4]

[1]Department of Biomedical Engineering
[2]Department of Medicine, Division of Infectious Diseases and International Health
[3]Department of Pathology
University of Virginia, Charlottesville, Virginia, USA

[4]Corresponding author
E-mail: papin@virginia.edu
Phone: 434.924.8195
Fax: 434.982.3870
Mail: Box 800759, Health System, University of Virginia, Charlottesville, Virginia, 22908, USA

Author e-mail addresses:
AKC: arvind@email.virginia.edu
ASB: asb9v@virginia.edu
JLT: jlt4m@virginia.edu
PAJ: paj2s@virginia.edu
RDP: rdp9g@virginia.edu
JP: papin@virginia.edu

Supplementary material: Additional file 1

In this supplement, additional data, analysis and network characteristics are presented that are not already described in the main article.

Supplementary text

Sensitivity of druggability, abnormal growth, FVA and STITCH confidence cutoffs

The sensitivity associated with various filters/cutoffs that were used in MetDP was explored (see supplementary Figure S1). The metrics that were varied include: (a) druggability index (panel A), (b) abnormal growth cutoff (panel B), (c) FVA score for genes (panel C), and (d) STITCH confidence (all panels). In every panel in supplementary Figure S1, the output includes the number of priority *L. major* genes (center of the bubble relative to the y-axis) and number of FDA-approved drugs (area of the bubble) for various STITCH confidence scores.

First, in supplementary Figure S1 panel A, the druggability index cutoff (x-axis) was varied from 0.1 (lowest) to 1 (highest). It should be noted that the abnormal growth cutoff and FVA score for genes were held constant at >30% and 1, respectively. Across all druggability scores, the number of priority *L. major* genes selected is constant as the STITCH confidence score is varied from 0.15 to 0.7; however, at the highest STITCH confidence (0.9), slight variations in numbers of *L. major* genes are evident at various druggability scores less than 0.7. Overall, the number of priority *L. major* genes varies from 22 to 2 as the druggability index increases from 0.1 to 1. Moreover, the number of FDA-approved drugs selected varies considerably across not only the druggability axis but also across the STITCH confidence of interactions. As illustrated, lower STITCH confidences result in a higher number of interactions between *L. major* genes and drugs. For each STITCH confidence, the numbers of *L. major* genes and associated FDA-approved drugs is constant as the druggability metric is varied from 0.3 to 0.6, and this range was thus considered to be 'moderate'. A druggability cutoff of 0.6 (as indicated by dotted line) was chosen. Additionally, at a druggability cutoff of 0.6, the percentage of true positives was 85%, 69%, 62% and 46% for STITCH confidence scores of >0.15, >0.4, >0.7 and >0.9, respectively. Based on these results, a moderate STITCH confidence score of >0.4 (as indicated by arrow) was selected.

Second, in supplementary Figure S1 panel B, the cutoff for the simulated growth rate as a percentage of wild type was varied across the x-axis (>0% defect, >30% defect or 100% defect). The druggability index and FVA score for genes were held constant at 0.6 and 1, respectively. For each STITCH confidence, the numbers of priority *L. major* genes and associated FDA-approved drugs are relatively constant across the various growth cutoffs. Hence, a moderate growth cutoff of >30% was chosen (as indicated by the dotted line).

Third, in supplementary Figure S1 panel C, the FVA score for genes was varied at three intervals (0.5, 0.75 and 1) while the druggability index and growth cutoffs were held constant (at 0.6 and >30%, respectively). As the FVA score is increased, the number of priority *L. major* genes decreases slightly for each STITCH confidence. For example, at a STITCH confidence >0.4, the number of priority *L. major* genes selected ranges from 22 (at an FVA score of 0.5) to 15 (at an FVA score of 1). Additionally, as the FVA cutoff is increased from 0.5 to 1, only modest decreases in the number of associated FDA-approved drugs are evident from the plot for each STITCH confidence. Since the FVA score for genes was specifically designed in this study (as

opposed to previously established metrics of druggability, FBA-based abnormal growth phenotype and STITCH confidence), a more stringent FVA cutoff of 1 was selected (as indicated by the dotted line).

Sensitivity of BLAST cutoffs

The sensitivity associated with target and drug predictions by varying the E-value cutoff (for BLAST-based sequence similarity between *L. major* genes and targets in DrugBank and STITCH) was also explored (see supplementary Figure S2). As shown in Figure 2 (of the main paper), an E-value cutoff of less than 0.001 (along with the selection of approved drugs and a STITCH confidence score of >0.4) resulted in 538 *L. major* genes being linked to 926 drugs. In contrast, an E-value cutoff of less than or equal to 1e-15 resulted in an initial association of 508 *L. major* genes and 887 drugs. Following other cutoffs included in MetDP, this initial E-value cutoff lead to the prioritization of 15 *L. major* targets and 182 FDA-approved drugs (see supplementary Figure S2). All nine true positives (those that have been used clinically against leishmaniasis; see supplementary Table S4) were included in the list of 182 drugs. Furthermore, an E-value cutoff of less than or equal to 1e-50 resulted in an initial association of 419 *L. major* genes and 658 drugs and a final prioritization of 15 *L. major* targets and 155 FDA-approved drugs. For this particular E-value cutoff, eight of the nine true positives were included in the list of 155 drugs (metronidazole was not present in the prioritized list). An E-value cutoff of less than 0.001 was chosen for the computational analysis in the paper because it allowed for a larger number of initial associations between *L. major* genes and drugs. These initial associations were subsequently refined via MetDP through target and drug selection.

Characteristics of priority drug targets and associated FDA-approved drugs

Of the priority drug targets (see Table 1 in the main paper), 11 had druggability scores greater than or equal to 0.8. Two genes, namely *LmjF25.1120* and *LmjF12.0280* encoding for aldehyde dehydrogenase and ornithine decarboxylase, had druggability scores of 1.0. And, with the exception of *LmjF25.1120*, the other 14 genes were predicted to be *in silico* essential. The fewest number of drugs associated with any one particular gene was three; two genes were associated with three drugs each. In contrast, one gene, namely *LmjF11.1100* encoding for sterol 14-demethylase, an enzyme participating in steroid biosynthesis, was linked to 133 drugs. Seven out of the 15 targets encoded for enzymes involved in steroid biosynthesis in the *L. major* reconstruction with corresponding reactions participating across multiple compartments including glycosome, cytosol, mitochondria and the endoplasmic reticulum. A total of 165 different drugs from Lm254 affect these seven targets highlighting the importance of steroid biosynthesis in terms of drug targeting against *L. major*. These seven target genes were essential to the metabolic model of *L. major* because ergosterol and zymosterol were unable to be synthesized as part of the biomass reaction (see Supplementary Figure S3). Sterols were included in biomass as part of the neutral lipid composition for *L. major* given that trypanosomatids have a strict requirement for particular endogenous sterols (e.g. ergosterol and other analogs) [1]. For generating the biomass reaction in the original model, it was

assumed that sterol and sterol esters in *L. major* were composed entirely of ergosterol and zymosterol [2].

Drugs in Lm254 were classified using several criteria namely, the Anatomical Therapeutic Chemical (ATC) classification system and Hodge and Sterner toxicity rating scale. The ATC classification system provides multi-level codes for drugs based on the anatomical system affected (first-level) and their therapeutic/chemical characteristics (second and higher levels), and these codes were derived from the DrugBank database. Drugs can have more than one code (e.g. aspirin) and some share the same code(s) (e.g. phenothiazine derivative drugs – promethazine and trimeprazine). Taking into account multiple yet distinct second-level codes per drug, one-third of the codes fell under three first-level categories: 'A' for alimentary tract and metabolism, 'C' for cardiovascular system and 'D' for dermatologicals. Within these first-level categories, a plurality of codes fell under second-level categories 'A01' (stomatological preparations), 'C10' (lipid modifying agents), and 'D01' (antifungals). Other prominent second-level categories included 'L01' (antineoplastic agents), 'S01' (ophthalmologicals), 'J01' (antibacterials) and 'G01' (gynecological antiinfectives and antiseptics). Together, these seven second-level categories (out of 73) account for nearly one-third of all codes that are associated with Lm254. A more in depth look at some of the categories overrepresented in Lm254 yields interesting insight on classes of drugs predicted to be active against *Leishmania* parasites. For example, many azole 'antifungals' such as ketoconazole and fluconazole have shown promise as antileishmanial agents and have been clinically evaluated in patients with leishmaniasis [3, 4].

Under the Hodge and Sterner toxicity rating classification (see Supplementary Table S1), the percentage of drugs with ratings of 6, 5, 4 and 3 were 2.4%, 15.0%, 53.1% and 29.5%, respectively. As outlined in the main paper, drugs with ratings below 3 signifying very high toxicity were excluded from consideration. Well-known drugs in Lm254 and that fall under these various rating categories include acyclovir (common anti-viral used in the treatment of herpes simplex virus infections; toxicity rating 6), atorvastatin (HMG-CoA reductase inhibitor; toxicity rating 5), acetaminophen (analgesic and antipyretic; toxicity rating 4) and aspirin (analgesic, antipyretic and anti-inflammatory medication; toxicity rating 3).

Excluding the 14 drugs in Lm254 prioritized via synthetic lethality analysis (considering only the 240 drugs acting on single lethal or growth-reducing targets), 80 are 'multi-functional' drugs acting on two or more targets (from the prioritized set in Table 1 of the main paper) while the remaining 160 are 'monofunctional' drugs acting on only one of 14 targets (*LmjF31.2940* encoding for squalene synthase is associated with only 'multifunctional' drugs). Of the 80 that are multifunctional, 52 are bifunctional and 20 are trifunctional. Of the remaining eight, seven act on four targets while one (lovastatin) is predicted to interact with six prioritized *L. major* genes. Importantly, *L. major* genes not included in the prioritized set of 15 were not considered in this analysis. Even still, it can be seen that many drugs are predicted to promiscuously target multiple *L. major* genes.

Target validation: false negative results

Of the 560 genes in the *L. major* metabolic reconstruction, 8 genes were associated with a perfect druggability score of 1.0. Of these, only two (*LmjF12.0280* encoding for ornithine decarboxylase; and *LmjF25.1120* encoding for aldehyde dehydrogenase) were present in the list of 15 prioritized targets. Of the remaining six targets with a perfect druggability score, three (*LmjF06.0860* encoding for dihydrofolate reductase-thymidylate synthase; and *LmjF22.1290* and *LmjF27.2050* together encoding for ribonucleoside-diphosphate reductase small chain) were a part of the 8 prioritized synthetic lethal targets. Finally, the last three genes with a druggability score of 1.0 (*LmjF24.1630* encoding for succinate dehydrogenase; *LmjF28.0890* encoding for ribonucleoside-diphosphate reductase large chain; and *LmjF19.1560* encoding for inosine-5'-monophosphate dehydrogenase) comprise the set of false negative targets that were not prioritized in either the single gene deletion or the synthetic lethality screens of MetDP. *LmjF24.1630* and *LmjF28.0890* were both essential *in silico*, but were associated with low FVA scores. And, *LmjF19.1560* was predicted to be non-essential to the metabolic network of *L. major*.

Drug validation: false negative results

Of the list of drugs that have been clinically evaluated against leishmaniasis (see main paper and supplementary Table S4), there were four false negative results: imiquimod [5], paromomycin [5-7], pentamidine [5-8] and sodium stibogluconate [4-8]. Pentamidine and imiquimod did not map to any *L. major* gene via BLAST-based sequence similarity to corresponding targets in DrugBank. However, through mapping using the STITCH database, subsequent to passing the druggability cutoff, both drugs were associated with *L. major* genes that were non-essential (0% growth defect). Therefore, imiquimod and pentamidine were effectively excluded at the 5th cutoff relating to abnormal growth phenotype (see Figure 2 of the main paper). Paromomycin was associated with a non-protein target in DrugBank (16S rRNA). And, although the drug was linked to a protein target in STITCH database, it did not map to any of the 560 *L. major* genes from the metabolic reconstruction via BLAST-based target similarity. Likewise, sodium stibogluconate (Pentostam) did not map to *L. major* genes via BLAST-based target similarity to corresponding targets in either DrugBank or STITCH databases. However, relaxing the STITCH confidence score (to greater than 0.15 instead of 0.4) allowed for paromomycin and sodium stibogluconate to be mapped to several *L. major* genes. Hence, paromomycin and sodium stibogluconate were excluded at the 3rd cutoff relating to STITCH confidence of interactions (see Figure 2 of the main paper).

In addition, of the list of compounds that were evaluated in the high-throughput screening study from Sharlow et al., 8 FDA-approved drugs (fluspirilene, terfenadine, astemizole, prochlorperazine, haloperidol, nitroxoline, chloroxine and nimodipine) were considered to be hits that were not included in Lm254.

Dependency on confidence of interactions in DrugBank and STITCH

Upon initial implementation of the MetDP pipeline, allopurinol was not present in the list of true positive candidates. Allopurinol is associated with xanthine oxidase (from *Homo sapiens*) in DrugBank, an enzyme that is lacking in trypanosomatids. Moreover, trypanosomatid species also lack the ability to synthesize purines *de novo*. Allopurinol, a structural isomer of hypoxanthine, acts as a purine analog and is incorporated via the enzyme hypoxanthine-guanine phosphoribosyltransferase (HGPRT). In contrast to DrugBank, the STITCH database lists HGPRT (in addition to xanthine oxidase and other targets) as a functional partner of allopurinol (CID000083786) with a high confidence score. However, compound CID000083786 was filtered out in the course of implementing MetDP, specifically during the STITCH database refinement process. This filtering occurred because DrugBank listed the PubChem compound identifier of allopurinol as 2094, which was used to map for the appropriate compound in STITCH (CID000002094). In STITCH, CID000002094 is annotated as the sodium form of allopurinol, which is not associated with HGPRT. Therefore, due to this mismatch, allopurinol initially did not map to any *L. major* gene from either DrugBank or STITCH analysis. Iterating through the pipeline, CID000002094 was manually replaced with CID000083786, which resulted in allopurinol being included in the list of clinically relevant positive candidates. Although, the *L. major* gene for HGPRT was filtered out due to a low FVA score, allopurinol was associated with another gene encoding for methylthioadenosine phosphorylase, which successfully passed the various cutoff metrics. This example of allopurinol is provided to highlight the dependence of the model and pipeline approach on the annotations (and confidences) of interactions in the datasets used.

Additional Notes

Composition of media (Complete HOMEM)

Minimal Essential Medium (10x) (Gibco 11430; Earle's salts without L-glutamine; Without $NaHCO_3$); MEM Amino Acids (50x) (Gibco 11130-051); MEM Non-essential amino acids (100x) (Gibco 11140); Sodium pyruvate (100x) (Gibco 11360); Glucose; Sodium bicarbonate; Biotin (Sigma B4639); Para amino benzoic acid (PABA) (Sigma A9878); Hepes buffer (1 M) (Gibco 15630); Gentamicin (50 mg/ml) (Cellgro 30-005-CR); L- glutamine (200mM) (Gibco 25030); Biopterin; Fetal calf serum (10%); Hemin (Sigma H5533)

Composition of Hepes Buffered Saline (HBS)

HEPES (21mM); Disodium hydrogen phosphate – Na_2HPO_4 (0.7mM); Sodium chloride – NaCl (137mM); Potassium chloride – KCl (5mM); Glucose (6mM)

LD50 data for toxicity analysis

To be consistent, data on LD50 data for rat under oral administration was culled for all the drugs. When such data was unavailable, a "best guess" toxicity rating was derived using data

from other animals (e.g. mouse, rabbit) tested under alternative means of administration (e.g. IP (intraperitoneal), IV (intravenous), SC (subcutaneous) and IM (intramuscular)). In a few cases, if the toxicity rating for certain drugs was judged to be higher than 2, they were automatically assigned a rating of 3 (lowest rating to pass toxicity and tolerance cutoff) as opposed to a higher rating. LD50 data for a few drugs was not readily available, and these compounds were filtered out from further analysis. See supplementary Table S2 for the list of all drugs in Lm254 and corresponding toxicity rating.

Supplementary figures and data

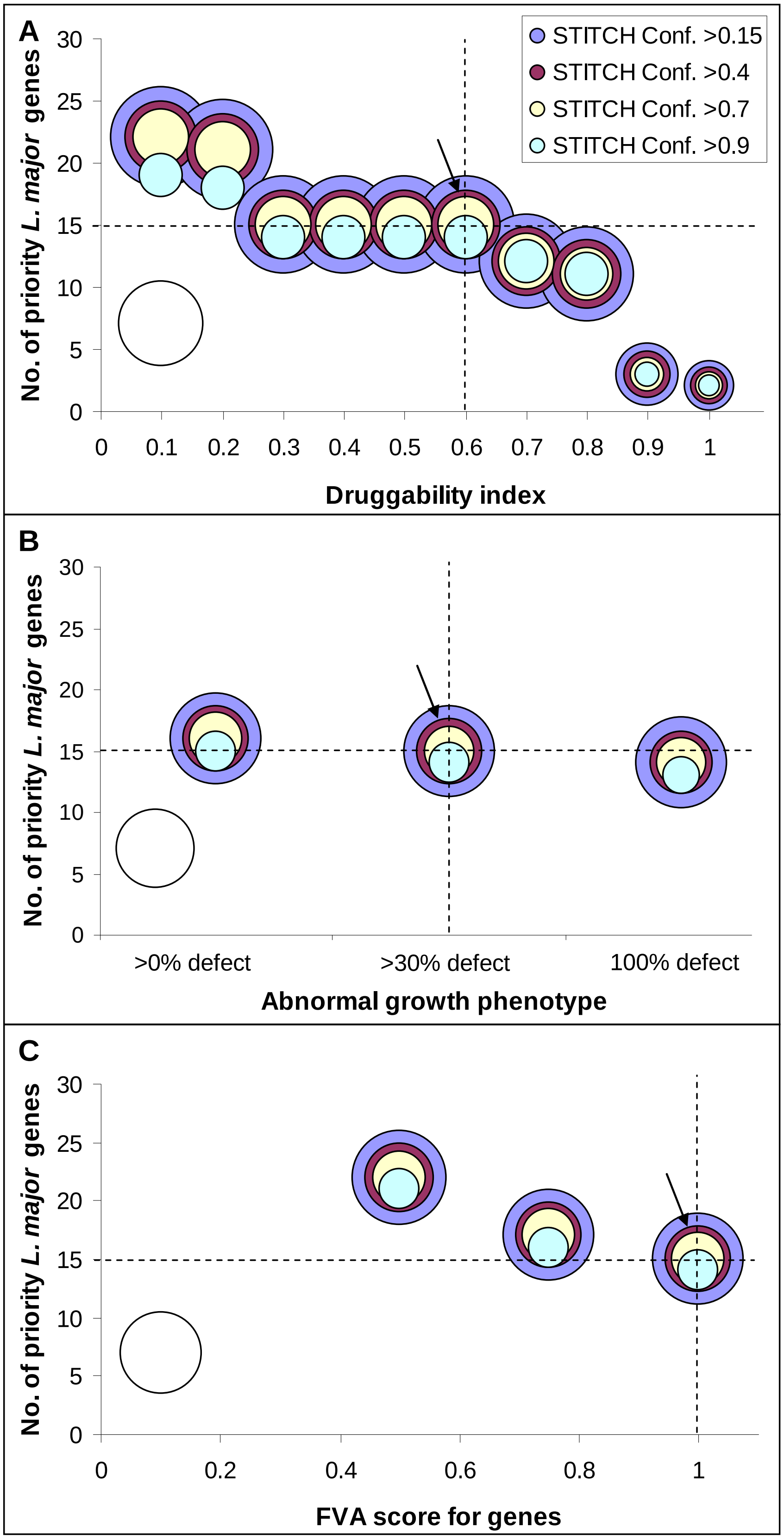

Sensitivity analysis of various cutoffs used in MetDP

Supplementary Figure S1: Sensitivity of cutoffs. The output includes the number of priority L. major genes (y-axis) and number of FDA-approved drugs (area of bubble) for various STITCH confidence scores. The empty circle (area set to 500 drugs) in the bottom left corner of each of the panels is provided for scale. Panel A: the druggability index cutoff (x-axis) was varied from 0.1 (lowest) to 1 (highest). Panel B: The cutoff for the simulated growth rate as a percentage of wildtype was varied across the x-axis (any defect, >30% defect or 100% defect). Panel C: The FVA score for genes was varied at three intervals (0.5, 0.75 and 1).

BLAST cutoff: E-value <= 1e-15

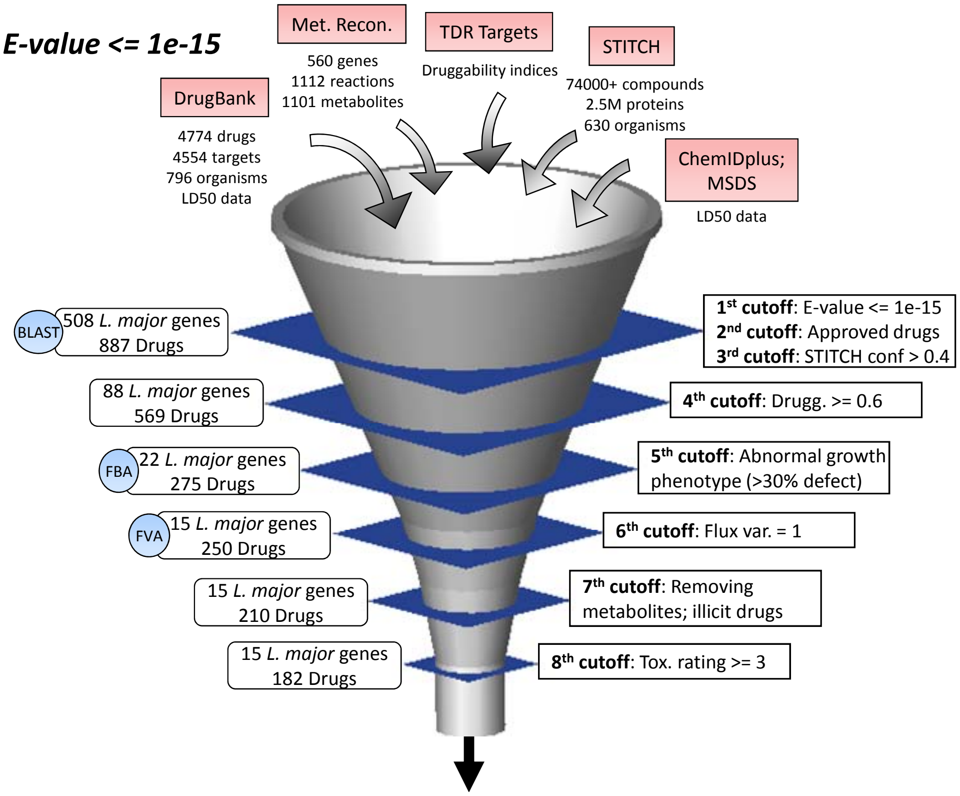

BLAST cutoff: E-value <= 1e-50

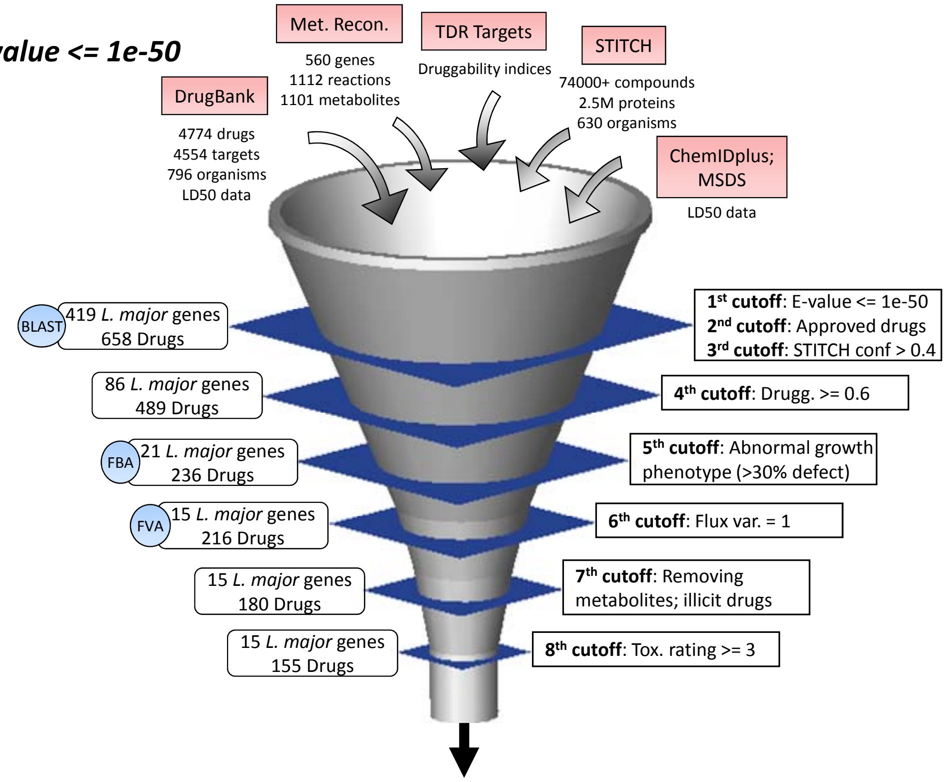

Supplementary Figure S2: Numbers of L. major *targets and drugs as determined via the MetDP scheme based on initial E-value cutoffs of <= 1e-15 and 1e-50.*

Priority drug targets and their effect on biomass production

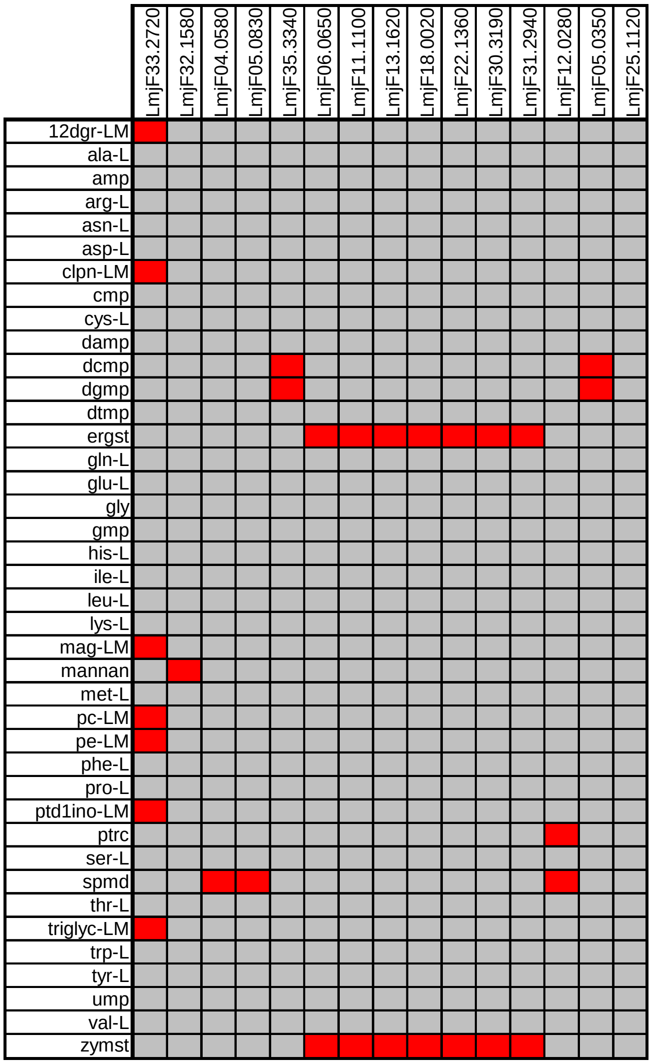

Supplementary Figure S3: Red boxes indicate metabolites in biomass that are unable to be synthesized given a knockout of a specific target gene in L. major*. See Table 1 in the main text for enzyme names corresponding to the targets presented in the figure. This analysis was performed by creating individual demand reactions for each of the biomass constituents.*

Hodge and Sterner toxicity classification

Toxicity rating	Commonly used term	Routes of Administration			Probable Lethal Dose for Man
		Oral LD50 (single dose to rats) mg/kg	Inhalation LC50 (exposure of rats for 4 hours) ppm	Dermal LD50 (single application to skin of rabbits) mg/kg	
1	Extremely Toxic	1 or less	10 or less	5 or less	1 grain (a taste, a drop)
2	Highly Toxic	1-50	10-100	5-43	4 ml (1 tsp)
3	Moderately Toxic	50-500	100-1000	44-340	30 ml (1 fl. oz.)
4	Slightly Toxic	500-5000	1000-10,000	350-2810	600 ml (1 pint)
5	Practically Non-toxic	5000-15,000	10,000-100,000	2820-22,590	1 litre (or 1 quart)
6	Relatively Harmless	15,000 or more	100,000	22,600 or more	1 litre (or 1 quart)

**Toxicity Classes: Hodge and Sterner Scale (http://www.ccohs.ca/oshanswers/chemicals/ld50.html)

Supplementary Table S1: Hodge and Sterner toxicity classification scheme

List of drugs in Lm254

All drugs in Lm254							
**Compound**	**Tox. Rating**	**Compound**	**Tox. Rating**	**Compound**	**Tox. Rating**	**Compound**	**Tox. Rating**
Acetaminophen	4	Dantrolene	5	Lansoprazole	5	Pravastatin	5
Acetylcysteine	5	Debrisoquin	4	Letrozole	3	Praziquantel	4
Aciclovir	6	Deferoxamine	4	Leucovorin	5	Primaquine	3
Albendazole	4	Dexamethasone	4	Lidocaine	3	Probucol	5
Alendronate	4	Dextromethorphan	3	Lindane	3	Procarbazine	4
Alitretinoin	4	Diazoxide	4	Lipoic Acid	3	Progesterone	5
Allopurinol	3	Diclofenac	3	Lithium	3	Propofol	4
Alprostadil	3	Dicumarol	3	Lomustine	3	Propranolol	3
Amantadine	4	Didanosine	4	Losartan	4	Propylthiouracil	4
Amikacin	4	Diltiazem	4	Lovastatin	4	Pyrazinamide	3
Aminocaproic Acid	6	Dimenhydrinate	4	Mannitol	4	Pyruvic acid	3
Aminoglutethimide	4	Dinoprostone	4	Masoprocol	4	Quinidine	3
Aminophylline	3	Diphenhydramine	4	Meclizine	4	Quinine	3
Amiodarone	4	Disulfiram	5	Mefloquine	4	Repaglinide	4
Amitriptyline	3	Docetaxel	4	Melatonin	4	Riboflavin	5
Amlodipine	3	Dopamine	4	Menadione	4	Rifampin	4
Amphotericin B	5	Doxorubicin	4	Mephenytoin	4	Risedronate	4
Ampicillin	5	Doxycycline	3	Mercaptopurine	3	Rosiglitazone	4
Antipyrine	4	Econazole	4	Metformin	4	Salicyclic acid	4
Aspartame	5	Epirubicin	4	Methimazole	4	Scopolamine	4
Aspirin	3	Erythromycin	4	Methoxsalen	4	Sertaconazole	5
Atorvastatin	5	Estriol	4	Metronidazole	4	Sertraline	4
Atropine	4	Ethacrynic acid	4	Metyrapone	4	Silver sulfadiazine	5
Auranofin	3	Ethinyl Estradiol	4	Miconazole	4	Simvastatin	4
Azelaic Acid	5	Etoposide	3	Mycophenolic acid	3	Spermine	3
Benzocaine	4	Ezetimibe	4	N-Acetyl-D-glucosamine	3	Streptomycin	3
Betamethasone	4	Felodipine	4	Naftifine	3	Streptozocin	5
Bezafibrate	4	Fenofibrate	4	Naloxone	4	Sulfacetamide	6
Bicalutamide	4	Fluconazole	4	Naproxen	3	Sulfamerazine	6
Bifonazole	4	Flucytosine	6	Nelfinavir	5	Sulindac	3
Bromocriptine	4	Fludarabine	3	Netilmicin	5	Tacrolimus	3
Bupropion	3	Fluocinolone Acetonide	4	Niacin	5	Tamoxifen	4
Buspirone	3	Fluorouracil	3	Nicardipine	3	Tazobactam	5
Butenafine	4	Flurbiprofen	3	Nifedipine	4	Telithromycin	4
Butoconazole	4	Flutamide	4	Nitric Oxide	3	Terazosin	3
Caffeine	3	Fluvastatin	4	Nitrofurantoin	4	Terbinafine	4
Carbamazepine	4	Fluvoxamine	3	Nitrofurazone	4	Terconazole	4
Cefepime	4	Fomepizole	4	Nitroglycerin	3	Testosterone	4
Ceftazidime	4	Furazolidone	4	Nitroprusside	3	Testosterone Propionate	4
Ceftriaxone	5	Ganciclovir	4	Nortriptyline	4	Tetracycline	4
Cefuroxime	5	Gemfibrozil	4	Nystatin	5	Tetrahydrofolic acid	3
Celecoxib	4	Glibenclamide	6	Omeprazole	4	Theophylline	3
Cerulenin	4	Guanabenz	3	Orphenadrine	3	Thiabendazole	4
Chloramphenicol	4	Guanidine	3	Paclitaxel	3	Thiamine	4
Chloroquine	4	Hydrocortisone	5	Pamidronate	4	Thioguanine	3
Chlorpromazine	3	Hydroxyurea	5	Pantoprazole	4	Tioconazole	4
Chlorpropamide	4	Hyoscyamine	3	Pargyline	3	Tolbutamide	4
Chlorzoxazone	4	Ibandronate	4	Pemetrexed	4	Tolnaftate	5
Cimetidine	5	Ifosfamide	3	Pentostatin	3	Tretinoin	4
Cladribine	3	Imipenem	3	Permethrin	4	Trimethoprim	5
Clarithromycin	4	Imipramine	3	Perphenazine	3	Tripelennamine	3
Clodronate	4	Indinavir	5	Phenoxybenzamine	4	Troleandomycin	5
Clofibrate	4	Irinotecan	4	Phenylbutazone	3	Ursodeoxycholic acid	5
Clonidine	3	Isoflurane	5	Phenylephrine	3	Valproic Acid	4
Clopidogrel	4	Isoniazid	3	Phenytoin	4	Verapamil	3
Clotrimazole	4	Isoproterenol	4	Pimozide	4	Vidarabine	5
Colestipol	4	Isotretinoin	4	Pioglitazone	3	Vitamin A	4
Cyclophosphamide	3	Itraconazole	3	Piperacillin	5	Vitamin E	4
Cysteamine	4	Kanamycin	4	Piroxicam	3	Voriconazole	3
Cytarabine	5	Ketoconazole	3	Posaconazole	5	Warfarin	3
**Drugs selected with synthetic lethality constraint**							
Capecitabine	4	Gemcitabine	4	Minocycline	4	Sucralfate	5
Floxuridine	3	Halofantrine	4	Nalidixic Acid	4	Trifluridine	4
Fluticasone Propionate	4	Histamine Phosphate	4	Novobiocin	4		
Framycetin	4	Levodopa	4	Probenecid	4		

Supplementary Table S2: List of drugs in Lm254. Drugs classified as "hits" are highlighted. The corresponding toxicity rating for the drugs is also provided.

Colour	Meaning
(orange)	Novel hit via *in vitro* analysis from this study
(light green)	Evaluated clinically against leishmaniasis
(pink)	Primary hit from Sharlow et al. HTS study
(grey-green)	Evaluated clinically and primary hit in Sharlow et al. study
(peach)	Primary hit from *T. brucei* HTS study
(light blue)	Evaluated *in vitro* in this study or in HTS screen, but no effect

Synthetic lethal predictions and associated FDA-approved drugs

Gene 1	Druggability	Drugs	Gene 2	Druggability	Drugs
LmjF27.2050	1	Cytarabine; Hydroxyurea; Acetaminophen; Gemcitabine; Epirubicin; Methoxsalen; Deferoxamine; Doxorubicin; Capecitabine; Cladribine; Nitric Oxide; Fluorouracil; Pentostatin;	LmjF22.1290	1	Cytarabine; Hydroxyurea; Acetaminophen; Gemcitabine; Epirubicin; Methoxsalen; Deferoxamine; Doxorubicin; Capecitabine; Cladribine; Nitric Oxide; Fluorouracil; Pentostatin;
LmjF06.0860	1	Cefotaxime; Flucytosine; Sulfamerazine; Cyanocobalamin; Riboflavin; Idoxuridine; Ampicillin; Streptozocin; Trimethoprim; Cephalexin; Leucovorin; Oxacillin; Gentamicin; Spectinomycin; Cytarabine; Hydroxyurea; Sulfamethoxazole; Penicillin G; Sertaconazole; Sulfadoxine; Erythromycin; Aminosalicylic Acid; Dapsone; Sulfanilamide; Raltitrexed; Mefloquine; Sulfadiazine; Trifluridine; Gemcitabine; Betamethasone; Epirubicin; Chloramphenicol; Framycetin; Amikacin; Altretamine; Vincristine; Lamotrigine; Chloroquine; Amodiaquine; Pemetrexed; Tamoxifen; Nitrofurantoin; Tetracycline; Irinotecan; Nalidixic Acid; Metronidazole; Doxorubicin; Rifampin; Capecitabine; Atovaquone; Kanamycin; Halofantrine; Dexamethasone; Docetaxel; Tetrahydrofolic acid; Pyrimethamine; Doxycycline; Floxuridine; Triamterene; Quinine; Oxaliplatin; Fluorouracil; Prednisone; Daunorubicin; Etoposide; Quinidine; Fexofenadine; Isoniazid; Carboplatin; Mercaptopurine; Streptomycin; Primaquine; Proguanil; Trimetrexate; Paclitaxel; Imipenem;	LmjF21.1210	0.8	Cytarabine; Hydroxyurea; Trifluridine; Epirubicin; Zidovudine; Stavudine; Doxorubicin; Capecitabine; Floxuridine; Fluorouracil;
LmjF20.0100	0.8	Sucralfate; Sertaconazole; Sildenafil; Framycetin; Mannitol; Azacitidine; Pyruvic acid;	LmjF24.0850	0.8	Aspartame; Sucralfate; Ipratropium; Trifluridine; Framycetin; Quinacrine; Pyruvic acid;
LmjF24.0850	0.8	Aspartame; Sucralfate; Ipratropium; Trifluridine; Framycetin; Quinacrine; Pyruvic acid;	LmjF30.3380	0.5	Sucralfate; Sertaconazole; Sildenafil; Framycetin; Mannitol; Azacitidine; Pyruvic acid;
LmjF04.0960	0.5	Vidarabine; Cytarabine; Gemcitabine; Salicyclic acid; Pyruvic acid;	LmjF34.0110	0.8	Vidarabine; Gemcitabine; Cerulenin; Pyruvic acid;
LmjF30.3520	0.6	Vidarabine; Silver sulfadiazine; Fluticasone Propionate; Histamine Phosphate; Nalidixic Acid; Cysteamine; Minocycline; Probenecid; Cerulenin; Levodopa; Tetrahydrofolic acid; Pyruvic acid; Spermine; Lipoic Acid; Doxycycline; Nitric Oxide; Nitroglycerin;	LmjF30.3500	0.6	Vidarabine; Silver sulfadiazine; Fluticasone Propionate; Histamine Phosphate; Nalidixic Acid; Cysteamine; Minocycline; Probenecid; Cerulenin; Levodopa; Tetrahydrofolic acid; Pyruvic acid; Spermine; Lipoic Acid; Doxycycline; Nitric Oxide; Nitroglycerin;
LmjF05.0510	0.5	Novobiocin; Halofantrine;	LmjF05.0500	0.5	Novobiocin; Halofantrine;
LmjF25.1180	0.5	Progesterone; Kanamycin; Halofantrine;	LmjF25.1170	0.5	Progesterone; Kanamycin; Halofantrine;

Supplementary Table S3: Synthetic lethal gene combinations with an average druggability index greater than or equal to 0.5. Both genes in the combination needed to have moderate druggability indices of 0.5 or higher. A total of eight non-trivial lethal double gene combinations satisfied this criterion.

Clinically relevant true positives and false negatives as compared to Lm254

TRUE POSITIVES			
**Drug Name**	**Predicted target(s) in *L. major* metabolic reconstruction**	**Predicted enzyme(s)**	**Metabolic pathways affected in reconstruction**
Amphotericin B	LmjF11.1100	Sterol 14-demethylase	Steroid biosynthesis
	LmjF13.1620	Squalene monooxygenase	Steroid biosynthesis
Ketoconazole	LmjF11.1100	Sterol 14-demethylase	Steroid biosynthesis
	LmjF13.1620	Squalene monooxygenase	Steroid biosynthesis
	LmjF06.0650	lanosterol synthase	Steroid biosynthesis
	LmjF32.1580	Phosphomannose isomerase	Fructose and mannose metabolism
Fluconazole	LmjF11.1100	Sterol 14-demethylase	Steroid biosynthesis
	LmjF13.1620	Squalene monooxygenase	Steroid biosynthesis
	LmjF31.2940	Squalene synthase	Steroid biosynthesis
Clotrimazole	LmjF11.1100	Sterol 14-demethylase	Steroid biosynthesis
Itraconazole	LmjF11.1100	Sterol 14-demethylase	Steroid biosynthesis
	LmjF13.1620	Squalene monooxygenase	Steroid biosynthesis
	LmjF31.2940	Squalene synthase	Steroid biosynthesis
Metronidazole	LmjF11.1100	Sterol 14-demethylase	Steroid biosynthesis
Miconazole	LmjF11.1100	Sterol 14-demethylase	Steroid biosynthesis
	LmjF13.1620	Squalene monooxygenase	Steroid biosynthesis
Terbinafine	LmjF11.1100	Sterol 14-demethylase	Steroid biosynthesis
	LmjF13.1620	Squalene monooxygenase	Steroid biosynthesis
Allopurinol	LmjF05.0830	methylthioadenosine phosphorylase	Methionine metabolism
**FALSE NEGATIVES**			
**Drug Name**	**Reasons for exclusion**		
Pentamidine	Associated *L. major* gene non-essential *in silico*		
Imiquimod	Associated *L. major* gene non-essential *in silico*		
Sodium stibogluconate	Low confidence of interaction with *L. major* genes		
Paromomycin	Low confidence of interaction with *L. major* genes		
**DRUGS EXCLUDED FROM CONSIDERATION**			
**Drug Name**	**Reasons for exclusion**		
Meglumine antimoniate	Not present in DrugBank		
Miltefosine	Not present in DrugBank		
Sitamaquine	Not present in DrugBank		

Supplementary Table S4: Drugs that were previously evaluated clinically against leishmaniasis are classified as true positives (or false negatives) if present (or absent) from Lm254

Calibration analysis of alamarBlue assay on **L. major** *promastigotes*

AlamarBlue fluorescence measure vs. seed concentration of parasites/well at various incubation times: Parasite samples were prepared at six different concentrations ($1x10^5$ cells/mL, $5x10^5$ cells/mL, $1x10^6$ cells/mL, $2x10^6$ cells/mL, $3x10^6$ cells/mL and $5x10^6$ cells/mL) by direct counting via hemocytometer. Cell count was determined after fixation in 2% formaldehyde (in water) for five minutes at room temperature. Subsequently, in a black flat-bottom 96-well microtiter plate, 160 µL of parasite samples at each of the six specific concentrations were seeded in triplicate. Each of the sample wells was topped off with 20 µL of media such that the total volume equaled 180 µL. Heat-killed parasite samples (incubated at 60°C for at least 20 minutes) prepared at $1x10^6$ cells/mL were also seeded in triplicate (160 µL of sample + 20 µL of media). Additionally, three wells were seeded with 180 µL of media alone. The plate was first incubated at 26°C for 24 hours. Subsequently, 20 µL of alamarBlue dye was added to all control and experimental wells. Using a Gemini EM Microplate Spectrofluorometer, fluorescence was monitored at excitation/emission wavelengths of 544nm/590nm at 30 minutes, 4.5 hours, 9 hours, 18 hours, 24 hours, 48 hours and 72 hours post addition of dye to wells.

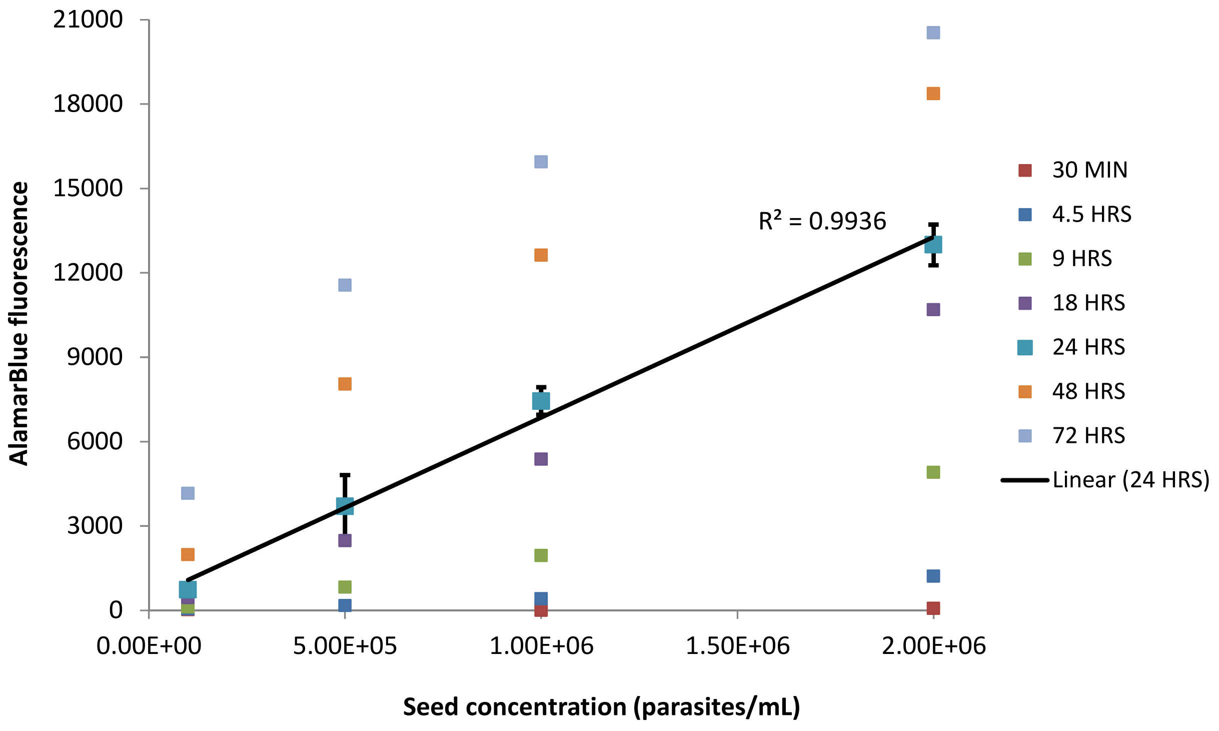

Supplementary Figure S4: Calibration of alamarBlue fluorescence vs. seed concentration of cells at various incubation times. Background fluorescence (with media alone) was subtracted. Figure shows linear regression analysis on data (for 24h time point). The R^2 value for is also indicated. The data points for $3*10^6$ and $5*10^6$ cells/mL are excluded as they do not fall within the linear range. Error bars signify standard deviation.

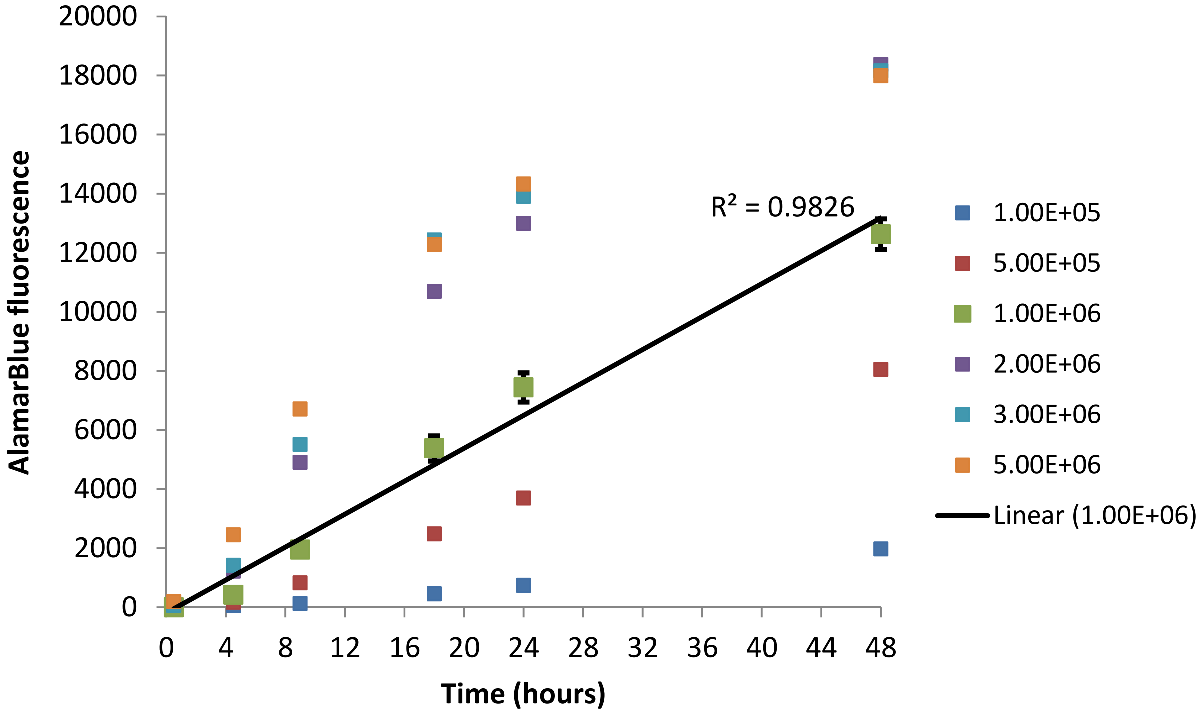

Supplementary Figure S5: Calibration of alamarBlue fluorescence vs. alamarBlue incubation times at various seed concentrations (cells/mL). Background fluorescence (with media alone) was subtracted. Figure shows linear regression analysis on data (for a seed concentration of $1*10^6$ *cells/mL). The* R^2 *value is also indicated. The data points for 72h are excluded as they do not fall within the linear range. Error bars signify standard deviation.*

Activity of Amphotericin B against* L. major *promastigotes as measured by alamarBlue fluorescence and manual cell counts

Parasite samples were prepared at 1x10^6 cells/mL by direct counting via hemocytometer. Cell count was determined after fixation in 2% formaldehyde (in water) for five minutes at room temperature. In a black flat-bottom 96-well microtiter plate, 160 µL of parasite samples were seeded in triplicate. Sample wells were topped off with 20 µL of media + Amphotericin B (ratio altered to achieve specific concentrations of drug) such that the total volume equaled 180 µL. Heat-killed parasite samples (incubated at 60°C for at least 20 minutes) prepared at 1x10^6 cells/mL were also seeded in triplicate (160 µL of sample + 20 µL of media). Additionally, three wells were seeded with 180 µL of media alone. The plate was first incubated at 26°C for 24 hours. Subsequently, 20 µL of alamarBlue dye was added to all control and experimental wells. Using a Gemini EM Microplate Spectrofluorometer, fluorescence was monitored at excitation/emission wavelengths of 544nm/590nm at 24 hours post addition of dye to wells. Manual readings were performed immediately. Cells were fixed at the time of counting with 2% formaldehyde.

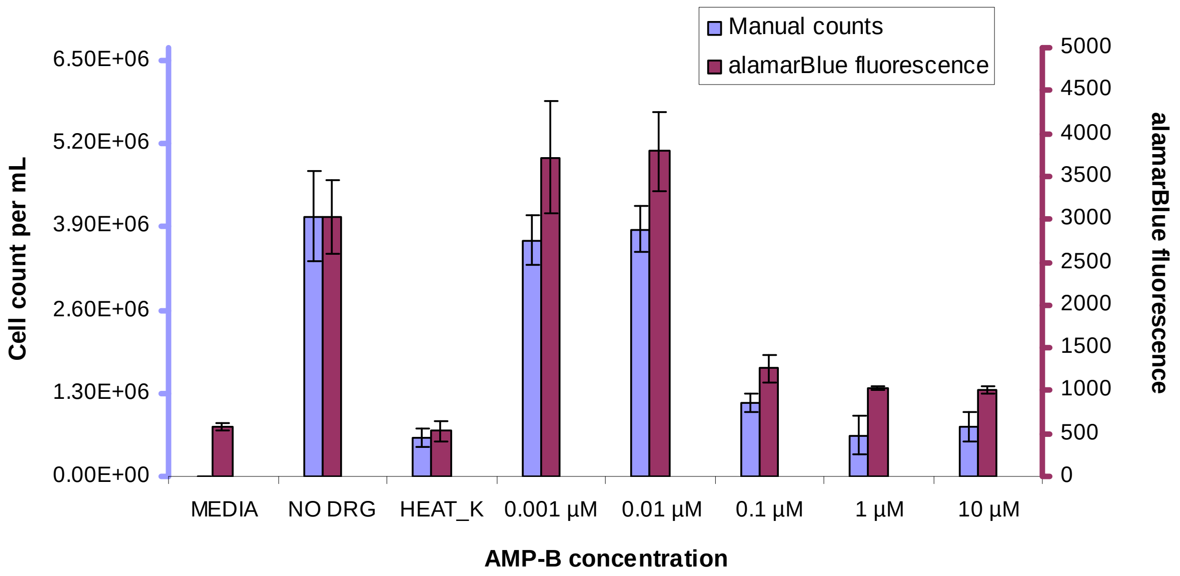

Supplementary Figure S6: Manual counts and alamarBlue fluorescence in assessing Amphotericin B activity against L. major. *The left y-axis represents manual cell counts per mL. The right y-axis represents alamarBlue fluorescence. The two axes were scaled such that the 'No Drug' control bars were of equal height. Plot demonstrates similar trend in the activity of Amphotericin B as measured by alamarBlue fluorescence or manual cell counts. Error bars signify standard deviation.*

Dose response data for disulfiram

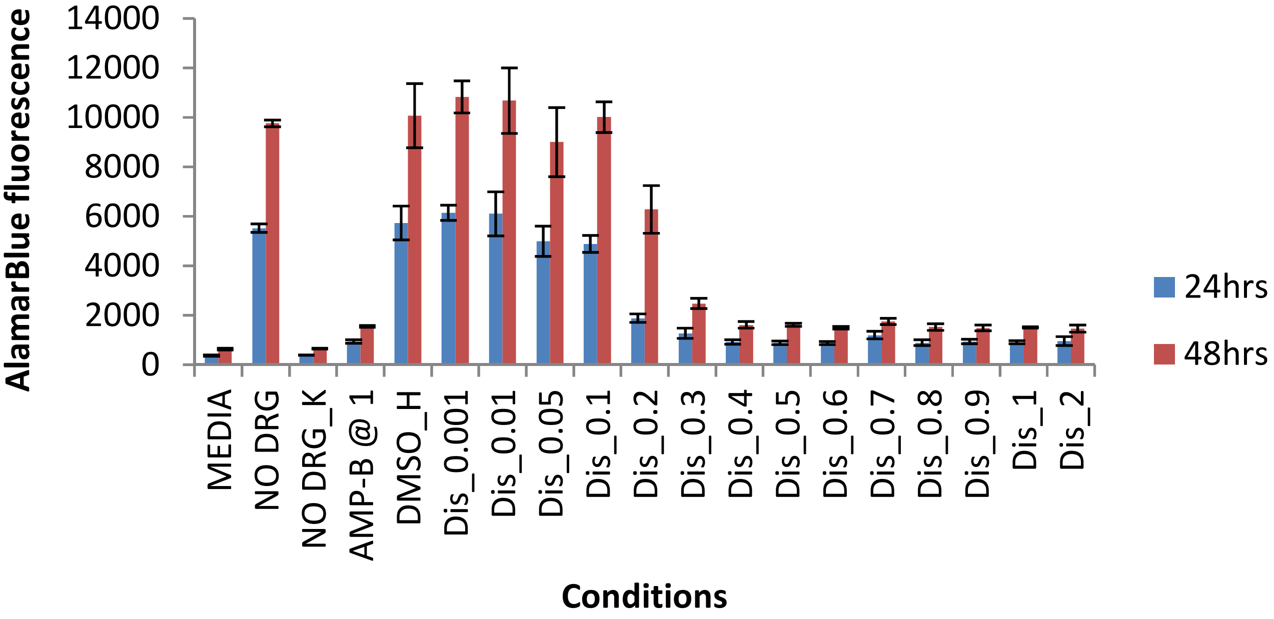

Supplementary Figure S7: Dose response data for disulfiram via the alamarBlue assay. The y-axis indicates alamarBlue fluorescence. All concentrations are in µM. Media is used as a control for background fluorescence. No Drug ('NO DRG') and DMSO at the highest relevant concentration ('DMSO_H') represent the negative controls. Heat-killed parasites ('NO DRG_K') and Amphotericin B at 1µM ('AMP-B @ 1') represent the positive controls. Error bars signify standard deviation.

Drugs combinations with disulfiram elucidated via synthetic lethality analysis

Gene #1	Enzyme name	Druggability	Drugs	Gene #2	Enzyme name	Druggability	Drugs	Avg. druggability
LmjF25.1180	F-type H+-transporting ATPase beta chain	0.5	Doxycycline, Clozapine, Nitric Oxide, Chlorpromazine, Isoniazid, Chloramphenicol, Framycetin, Metronidazole, Kanamycin, Clarithromycin, Halofantrine, Amoxicillin, Vidarabine, Progesterone, Disulfiram, Spectinomycin	LmjF25.1170	F-type H+-transporting ATPase beta chain	0.5	Doxycycline, Clozapine, Nitric Oxide, Chlorpromazine, Isoniazid, Chloramphenicol, Framycetin, Metronidazole, Kanamycin, Clarithromycin, Halofantrine, Amoxicillin, Vidarabine, Progesterone, Disulfiram, Spectinomycin	0.5

Supplementary Table S5: Synthetic lethality analysis with a relaxed STITCH confidence score identified drug combinations involving disulfiram

Additional experimental data for Disulfiram and Kanamycin

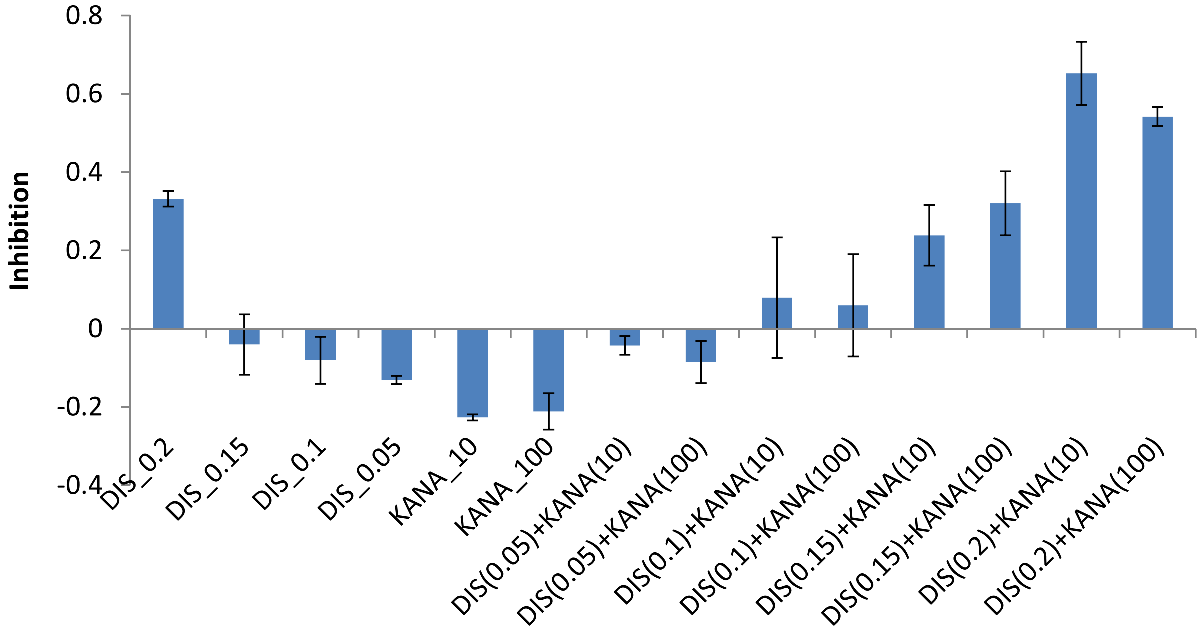

Supplementary Figure S8: Additional experimental data for disulfiram + kanamycin at 48 hours post addition of alamarBlue dye. Concentrations are in μM. Error bars signify standard error. The y-axis indicates fractional experimental effect of inhibition or growth relative to "No Drug" control (0 equals no inhibition, 1 equals max inhibition).

Additional experimental data for Disulfiram and Clozapine

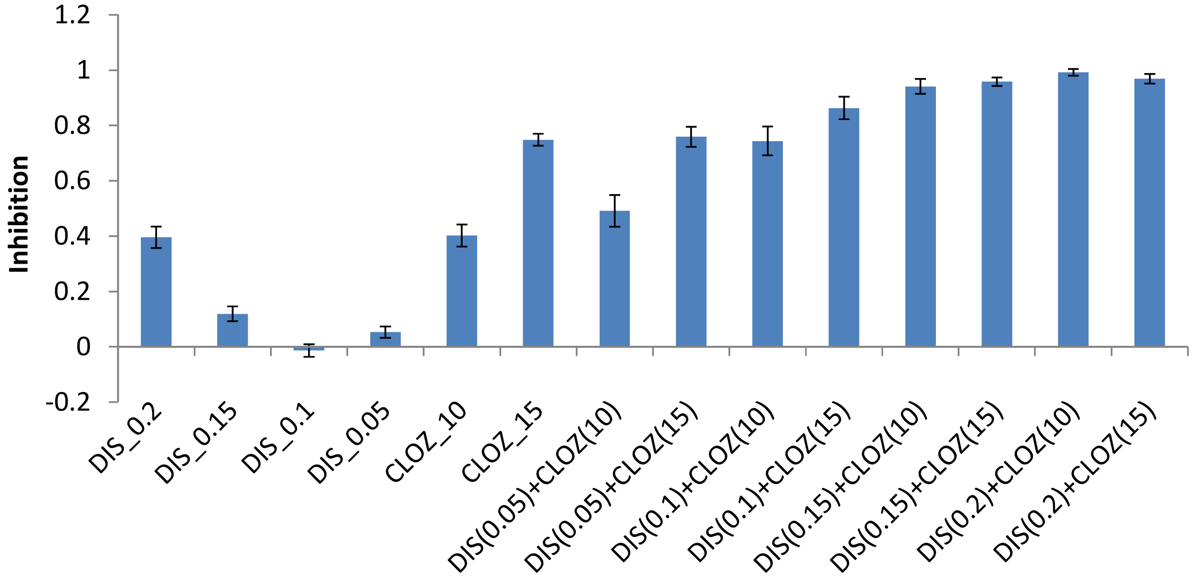

Supplementary Figure S9: Additional experimental data for disulfiram + clozapine at 48 hours post addition of alamarBlue dye. Concentrations are in µM. Error bars signify standard error. The y-axis indicates fractional experimental effect of inhibition or growth relative to "No Drug" control (0 equals no inhibition, 1 equals max inhibition).

Additional experimental data for Disulfiram and Amoxicillin

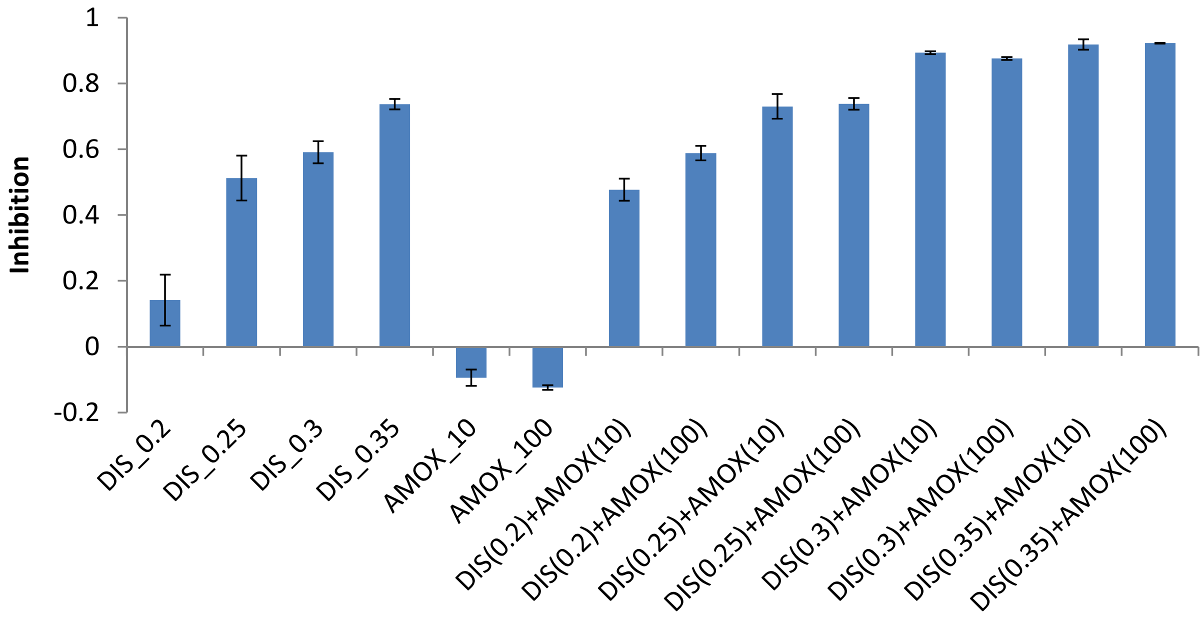

Supplementary Figure S10: Additional experimental data for disulfiram + amoxicillin at 48 hours post addition of alamarBlue dye. Concentrations are in µM. Error bars signify standard error. The y-axis indicates fractional experimental effect of inhibition or growth relative to "No Drug" control (0 equals no inhibition, 1 equals max inhibition).

Additional experimental data for Disulfiram and Chlorpromazine

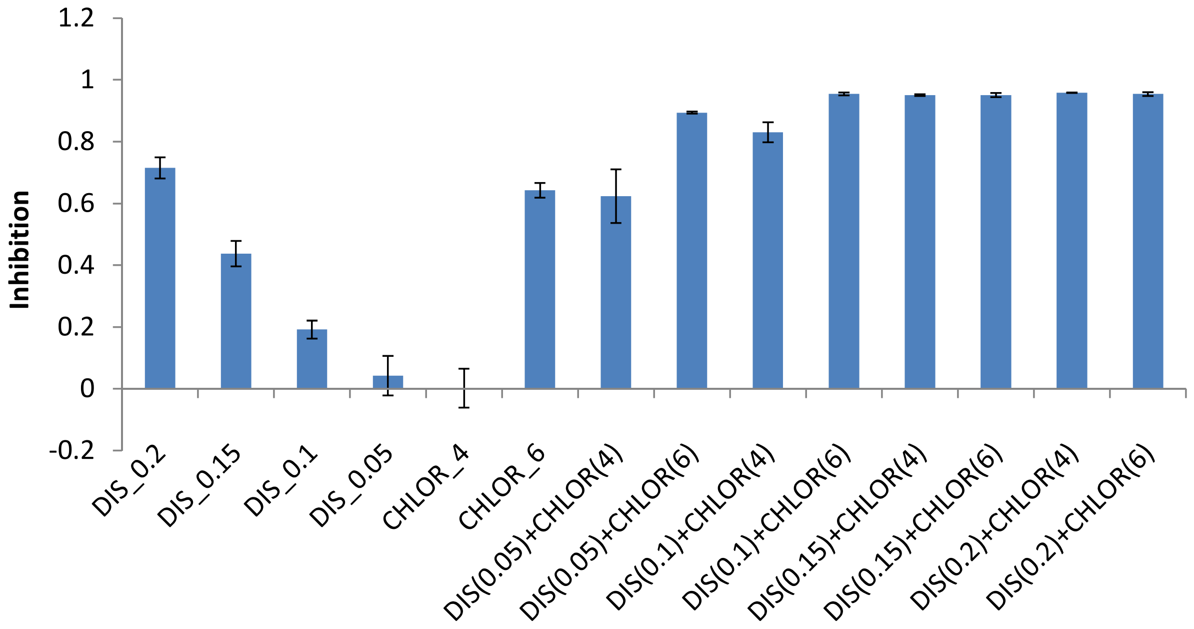

Supplementary Figure S11: Additional experimental data for disulfiram + chlorpromazine at 48 hours post addition of alamarBlue dye. Concentrations are in µM. Error bars signify standard error. The y-axis indicates fractional experimental effect of inhibition or growth relative to "No Drug" control (0 equals no inhibition, 1 equals max inhibition).

Calibration analysis of CellTiter-Glo assay on* L. major *promastigotes

Parasite samples were prepared at 12 different concentrations ($1x10^5$, $5x10^5$, $1x10^6$, $2x10^6$, $3x10^6$, $4x10^6$, $5x10^6$, $6x10^6$, $7x10^6$, $8x10^6$, $9x10^6$ and $1x10^7$ cells/mL) by direct counting via hemocytometer. Cell count was determined after fixation in 2% formaldehyde (in water) for five minutes at room temperature. Parasite samples were incubated at 26°C for 3 hours. Subsequently, in a white opaque flat-bottom 96-well microtiter plate, 25 µL of parasite samples at each of the 12 specific concentrations were seeded in triplicate. Heat-killed parasite samples (incubated at 60°C for at least 20 minutes) prepared at $8x10^6$ cells/mL were also seeded in triplicate. Additionally, three wells were seeded with 25 µL of media alone. Subsequently, 25 µL of CellTiter-Glo was added to all control and experimental wells. The plate was incubated in the dark at 26°C for 10 minutes. Luminescence was monitored using a FLUOstar Optima plate reader (BMG Labtech).

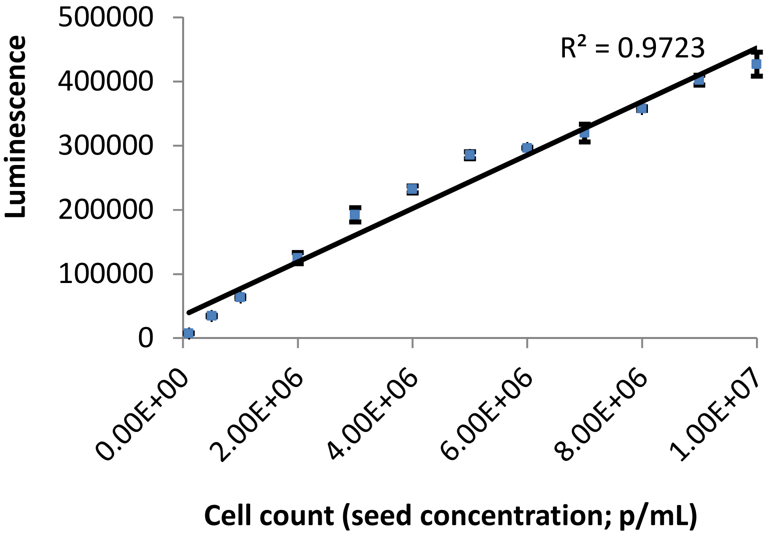

Supplementary Figure S12: Calibration of CellTiter-Glo luminescence vs. seed concentration of cells/well. Background luminescence (with media alone) was subtracted. Figure shows linear regression analysis on data. The R^2 value for is also indicated. Error bars signify standard deviation.

ATP standard curve

ATP was prepared at varying concentrations in cell culture medium (HOMEM). In a white opaque flat-bottom 96-well microtiter plate, 25 µL of ATP samples at each concentration were seeded in triplicate. Additionally, three wells were seeded with 25 µL of media alone. Subsequently, 25 µL of CellTiter-Glo was added to all control and experimental wells. The plate was incubated in the dark at 26°C for 10 minutes. Luminescence was monitored using a FLUOstar Optima plate reader (BMG Labtech).

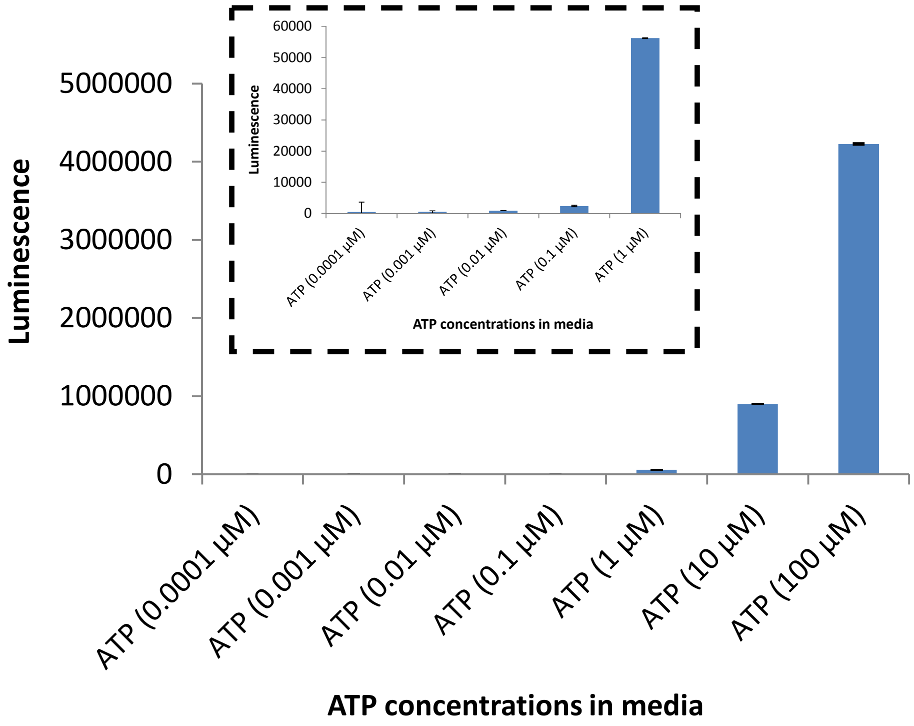

Supplementary Figure S13: ATP standard curve generated with CellTiter-Glo. Inset shows higher resolution of ATP concentrations not readily visible in the main plot. Error bars signify standard deviation.

References

1. Urbina JA, Concepcion JL, Rangel S, Visbal G, Lira R: **Squalene synthase as a chemotherapeutic target in Trypanosoma cruzi and Leishmania mexicana**. *Mol Biochem Parasitol* 2002, **125**(1-2):35-45.
2. Chavali AK, Whittemore JD, Eddy JA, Williams KT, Papin JA: **Systems analysis of metabolism in the pathogenic trypanosomatid Leishmania major**. *Mol Syst Biol* 2008, **4**:177.
3. Alrajhi AA, Ibrahim EA, De Vol EB, Khairat M, Faris RM, Maguire JH: **Fluconazole for the treatment of cutaneous leishmaniasis caused by Leishmania major**. *N Engl J Med* 2002, **346**(12):891-895.
4. Navin TR, Arana BA, Arana FE, Berman JD, Chajon JF: **Placebo-controlled clinical trial of sodium stibogluconate (Pentostam) versus ketoconazole for treating cutaneous leishmaniasis in Guatemala**. *J Infect Dis* 1992, **165**(3):528-534.
5. Mishra J, Saxena A, Singh S: **Chemotherapy of leishmaniasis: past, present and future**. *Curr Med Chem* 2007, **14**(10):1153-1169.
6. Blum J, Desjeux P, Schwartz E, Beck B, Hatz C: **Treatment of cutaneous leishmaniasis among travellers**. *J Antimicrob Chemother* 2004, **53**(2):158-166.
7. Singh S, Sivakumar R: **Challenges and new discoveries in the treatment of leishmaniasis**. *J Infect Chemother* 2004, **10**(6):307-315.
8. Olliaro PL, Bryceson AD: **Practical progress and new drugs for changing patterns of leishmaniasis**. *Parasitol Today* 1993, **9**(9):323-328.
